# Pyrene Functionalized Highly Reduced Graphene Oxide-palladium Nanocomposite: A Novel Catalyst for the Mizoroki-Heck Reaction in Water

**DOI:** 10.3389/fchem.2022.872366

**Published:** 2022-04-29

**Authors:** Mujeeb Khan, Muhammad Ashraf, Mohammed Rafi Shaik, Syed Farooq Adil, Mohammad Shahidul Islam, Mufsir Kuniyil, Merajuddin Khan, Mohammad Rafe Hatshan, Riyadh H. Alshammari, Mohammed Rafiq H. Siddiqui, Muhammad Nawaz Tahir

**Affiliations:** ^1^ Department of Chemistry, College of Science, King Saud University, Riyadh, Saudi Arabia; ^2^ Chemistry Department, King Fahd University of Petroleum and Minerals, Dhahran, Saudi Arabia; ^3^ Interdisciplinary Research Center for Hydrogen and Energy Storage (IRC-HES), King Fahd University of Petroleum and and Minerals, Dhahran, Saudi Arabia

**Keywords:** highly reduced graphene, palladium, catalyst, mizoroki-heck, aqueous synthesis

## Abstract

The formation of a C-C bond through Mizoroki-Heck cross-coupling reactions in water with efficient heterogeneous catalysts is a challenging task. In this current study, a highly reduced graphene oxide (HRG) immobilized palladium (Pd) nanoparticle based catalyst (HRG-Py-Pd) is used to catalyze Mizoroki-Heck cross-coupling reactions in water. During the preparation of the catalyst, amino pyrene is used as a smart functionalizing ligand, which offered chemically specific binding sites for the effective and homogeneous nucleation of Pd NPs on the surface of HRG, which significantly enhanced the physical stability and dispersibility of the resulting catalyst in an aqueous medium. Microscopic analysis of the catalyst revealed a uniform distribution of ultrafine Pd NPs on a solid support. The catalytic properties of HRG-Py-Pd are tested towards the Mizoroki-Heck cross-coupling reactions of various aryl halides with acrylic acid in an aqueous medium. Furthermore, the catalytic efficacy of HRG-Py-Pd is also compared with its non-functionalized counterparts such as HRG-Pd and pristine Pd NPs (Pd-NPs). Using the HRG-Py-Pd nanocatalyst, the highest conversion of 99% is achieved in the coupling reaction of 4-bromoanisol and acrylic acid in an aqueous solution in a relatively short period of time (3 h), with less quantity of catalyst (3 mg). Comparatively, pristine Pd NPs delivered lower conversion (∼92%) for the same reaction required a long reaction time and a large amount of catalyst (5.3 mg). Indeed, the conversion of the reaction further decreased to just 40% when 3 mg of Pd-NPs was used which was sufficient to produce 99% conversion in the case of HRG-Py-Pd. On the other hand, HRG-Pd did not deliver any conversion and was ineffective even after using a high amount of catalyst and a longer reaction time. The inability of the HRG-Pd to promote coupling reactions can be attributed to the agglomeration of Pd NPs which reduced the dispersion quality of the catalyst in water. Therefore, the high aqueous stability of HRG-Py-Pd due to smart functionalization can be utilized to perform other organic transformations in water which was otherwise not possible.

## Introduction

Traditionally, organic reactions are performed in hazardous organic solvents due to the higher solubility of most of the reactants in these mediums (Kitanosono et al., 2018). Since solvents are used in large quantities comparatively to reactants, currently they are considered the greater threat to the environment (Cseri et al., 2018). Therefore, in current circumstances, chemists are facing the challenging task of developing green technologies for important organic transformations (Soh and Eckelman, 2016). The “*green processes*” should be capable of performing organic reactions utilizing eco-friendly, sustainable, and economically beneficial conditions (Sheldon, 2018). In this regard, the application of water as a reaction medium greatly reduces the environmental impact of organic synthesis (Simon and Li, 2012). Due to its extraordinary characteristics such as biocompatibility, non-flammability and high vapor pressure, etc., water is considered “nature’s choice of solvent” (Gawande et al., 2013). In addition to the environmental and economic benefits, water allows mild reaction conditions, simplifies chemical processes, and also offers unique reactivity and selectivity (Butler and Coyne, 2010). Nowadays, water is largely explored as a safe and suitable alternative to the hazardous organic solvents for the preparation of various industrially important fine chemicals and pharmaceuticals compounds (Nebra and García-Álvarez, 2020).

Among these, biphenyl derivatives and acrylates are an important class of compounds that are extensively used as starting materials during the synthesis of several applied chemicals in the pharmaceutical and agrochemical fields. (Kambe et al., 2011). Generally, methods used for the synthesis of these compounds include Suzuki–Miyaura and Mizoroki–Heck cross-coupling reactions (Frisch and Beller, 2005; Khazaei et al., 2017; Baran and Nasrollahzadeh, 2020; Easson et al., 2020). The latter is the coupling of aryl halides with alkenes using palladium-based catalysts in the presence of a base (Amatore and Jutand, 2000; Khan et al., 2017). These types of coupling reactions are typically applied for the preparation of stilbenes and cinnamic acid types of useful natural products, pharmaceuticals, and other compounds with specific properties including the presence of trans double bond (De Filippis et al., 2017; Gao et al., 2019). Mostly, coupling reactions including the Suzuki–Miyaura coupling have a few downsides, like difficulty in the recovery of precious metals-based catalysts, application of costly ligands, and environmental hazards due to the use of organic solvents (Nasrollahzadeh et al., 2015; Guo et al., 2017). Therefore, the use of water as a reaction medium for Mizoroki–Heck reactions would greatly enhance the development of chemical industrial processes and also minimize the environmental impact (De Meijere and Meyer, 1995).

However, in most cases, water is not considered as an appropriate solvent for the Mizoroki–Heck reactions. These are usually carried out in organic solvents due to the relatively higher solubility of reactants involved in the reactions (Phan et al., 2006). Depending on the solubility of reaction components, effortshave been carried out to develop palladium-catalyzed coupling reactions including Mizoroki–Heck cross-coupling using water as solvent (Nasrollahzadeh et al., 2014b; Jagtap, 2017; Christoffel and Ward, 2018). For this purpose, inorganic salt promoters, the addition of phase-transfer agents, and the use of alternative energy sources like microwave or ultrasound are used to overcome the solubility challenge (Christoffel and Ward, 2018). Nevertheless, continued research in the field of aqueous chemistry of the Mizoroki–Heck reactions would be strongly beneficial to the environment (Jadhav and Rode, 2017).

Typically, coupling reactions are performed using palladium salts or complexes as homogeneous catalysts. However, homogeneous reactions usually suffer from the loss or contamination of residual catalysts and often require tedious workup procedures (Beletskaya and Cheprakov, 2000). To avoid this, Pd NPs are commonly used as catalysts because nanoparticles offer the benefits of both homogeneous (solubility) and heterogeneously (easy recovery and improved cyclability). Particularly, when synthesized using green protocols which are independent of hazardous ligands (Mpungose et al., 2018). The stability, separation, cyclability, and reactivity of Pd NPs can be further enhanced by immobilizing them on suitable solid supports (support materials in the nanosize regime) such as graphene or mesoporous silica nanoparticles (Kuniyil et al., 2019; Hong et al., 2020). Furthermore, these supports compensate for the high cost of palladium because the amount of used palladium in the main catalyst is diluted (Felpin et al., 2006). Therefore, the use of effective supports is highly appreciable (Alonso et al., 2018). Among various supports graphene offers a flat 2D surface with high surface area for better adsorption of reactants, greatest intrinsic carrier mobility, perfect atomic lattice, excellent mechanical strength, and promising chemical and thermal stability (Julkapli and Bagheri, 2015; Naghdi et al., 2016c; Nasrollahzadeh et al., 2018; Qian et al., 2020). In addition, graphene supports can be converted to both hydrophobic and hydrophilic substances with easy customization, which can be very useful in heterogeneous catalysis (Nasrollahzadeh et al., 2014a; Fakhri et al., 2015; Naghdi et al., 2016a; Naghdi et al., 2016b). Hence, the development of a graphene supported Pd based water dispersible catalyst which can be easily separated from the product is highly desirable.

Considering the importance of water in Pd catalyzed reactions, herein we report the synthesis of a water-dispersible, heterogeneous catalyst for the Mizoroki–Heck coupling of aryl halides and acrylic acid in an aqueous medium (cf. Scheme 1). The heterogeneous catalyst was prepared by immobilizing smaller size Pd NPs on pyrene functionalized highly reduced graphene oxide according to our previously reported method (Khan et al., 2020). The as-prepared catalyst is highly dispersible in water, stable over weeks, and can be easily separated from the reaction medium by simple filtration or centrifugation. Furthermore, to evaluate the importance of functionalization, the catalytic activity of pyrene functionalized highly reduced graphene oxide-palladium nanocomposite (HRG-Py-Pd) is compared with its non-functionalized counterpart (HRG-Pd) and pristine Pd nanoparticles.

## Experimental

### Materials and Methods

All reactants and solvents were purchased from commercial suppliers (Sigma-Aldrich) and used without any further purification (extra purified chemicals are specifically indicated in the main text). Graphite powder (99.999%, −200 mesh) was purchased from Alfa Aesar. Other materials used are 1-aminopyrene (1-AP, 97%), sodium tetrachloropalladate (II) (99.9%), concentrated sulfuric acid (H_2_SO_4_, 98%), potassium permanganate (KMnO_4_, 99%), sodium nitrate (NaNO_3_, 99%), hydrogen peroxide (H_2_O_2_, 30 wt%), Acrylic acid, n-butylacrylate, 4-bromoanisol, sodium dodecyl sulfate (SDS, 98%), K_3_PO_4_ etc. FT-IR spectra were measured on Perkin Elmer 1,000 FT-IR spectrometer from 400 to 4,000 cm^−1^ by using KBr pellets. ^1^H and ^13^C spectra were obtained on a JEOL JNM-ECP 400 NMR spectrometer. Powder XRD patterns were recorded on D2 Phaser X-ray diffractometer (Bruker, Germany), Cu Kα radiation (k = 1.5418 A°). High-resolution transmission electron microscopy (HRTEM) images and EDX were measured on JEM 2100F (JEOL, Tokyo, Japan)). HPLC analysis on a Shimadzu LC-20A Prominence instrument (Shi-madzu, Kuoto, Japan). Column used: Daicel Chiralcel OD-H columns (Chiral Technologies Europe, Illkirch Graffenstaden, France) (80–95% n-hexane/iso-propanol).

### Synthesis and Functionalization of HRG

Highly reduced graphene oxide was prepared according to our previously reported method (Khan et al., 2020). For the preparation of HRG graphene oxide was used as a precursor which was synthesized by the oxidation of graphite powder using a modified Hummers method (Hummers and Offeman, 1958; Cote et al., 2009). For the functionalization of HRG, freshly prepared HRG is functionalized by using 1-aminopyrene (1-AP) as a ligand. Briefly, a 10 ml dispersion of methanol was prepared using sonication (30 min) by adding 25 mg of HRG. Separately, 25 mg of 1-AP was dissolved in methanol (10 ml) using stirring at room temperature. Both mixtures were combined and stirred together for 48 h at room temperature. Thereafter, the resultant mixture was sonicated at 20 °C for 6 h. Finally, the sample was centrifuged for 15 min to get rid of residual 1-AP and the functionalized HRG is isolated. The sample was further purified by re-dispersing in fresh methanol (5 ml). The resulting dispersion was sonicated at 20°C (30 min), centrifuged for 1 hour and the product was isolated by simply decanting the resulting mixture. This process was repeated several times (3–4 times) to achieve maximum purity of the sample which is dried overnight under vacuum.

### Synthesis of HRG-Py-Pd, HRG-Pd, and Pristine Pd NPs

To prepare HRG-Py-Pd equivalent weight (1:1) of freshly prepared functionalized HRG and palladium precursor (Na_2_PdCl_4_) is used. For this purpose, 5 mg of 1-AP functionalized HRG was dispersed in 5 ml of ethanol which was added to a separately prepared 5 ml ethanol solution of Pd precursor (Na_2_PdCl_4,_ 5 mg, 0.0169 mmol). The mixture was sonicated for 1 h which resulted in the formation of the functionalized nanocomposite. Finally, the product was separated by centrifugation (9,000 rpm), which was re-dispersed in water (10 ml) for later use. The non-functionalized counterpart of HRG-Py-Pd was also prepared in a similar fashion, however in this case freshly prepared pristine HRG was used to obtain HRG-Pd. Pd NPs were also obtained using sonication in a similar procedure as described above. In a typical procedure, 10 mg of Na_2_PdCl_4,_ (0.0338 mmol) was dissolved in 10 ml of ethanol through stirring at room temperature. The solution was then sonicated for 1 h, subsequently, the product is isolated by centrifugation and redispersed in 10 ml ethanol and sonicated for further purification. The final product was separated by centrifugation.

### General Procedure For The Mizoroki-Heck Coupling Reaction in an Aqueous Medium

In an airtight screw-capped vial (5 ml) catalyst (HRG-Py-Pd, 3 mg) or (Pd-NPs, 5.3 mg), K_3_PO_4_ (106 mg, 0.5 mmol), SDS (72 mg, 0.5 eq., 0.25 mmol) and water (2 ml) were charged with a small magnetic stir bar. Then aryl halide **1** (0.5 mmol) was added to it followed by acrylic acid **2** (36 mg, 0.5 mmol). The reaction was left stirring at 100°C for 3 h. The reaction was monitored by HPLC (30% Hex/^*i*^PrOH, 0.5 ml/min). Then the vial was cooled to room temperature and the product was extracted in ethyl acetate (3 × 3 ml). The ethyl acetate layer was basified with aqueous 4M K_2_CO_3_ (5 ml) and separated from the organic layer then the aqueous layer was again acidified with 4N HCl (PH 1-2). Finally, the pure product was then extracted in ethyl acetate (3 × 5 ml) and the combined organics were dried over anhydrous MgSO_4_. The organic layer was then concentrated under reduced pressure to afford pure compounds MH product **3a-f.** All the products were characterized by ^1^H-NMR and ^13^C-NMR spectra.

### Large Scale Mizoroki-Heck Reaction

For this, 4-bromoanisol **1** (1.86 g, 10 mmol) and acrylic acid **2** (720 mg, 10 mmol) in the presence of a catalyst (HRG-Py-Pd, 30.0 mg) and (Pd-NPs, 53.0 mg) were reacted according to the general procedure described above (scale 20 times, but the time was doubled to 6 h) to afford pure **3a.** Catalyst HRG-Py-Pd yielded 1.73 g (97%) and Catalyst Pd-NPs yielded 1.58 g (89%) respectively.

**(*E*)-3-(4-methoxyphenyl)acrylic acid (3a):** m.p 171–173°C (lit.168–170°C)^1^; ^1^H-NMR (400 MHz, DMSO-*d*_6_) δ 12.23 (s, 1H, COO**H**), 7.64 (d, *J* = 8.8 Hz, 2H, Ar-**H**), 7.54 (d, *J* = 16.0 Hz, 1H, C**H** = CH), 6.97 (d, *J* = 8.2 Hz, 2H, Ar-**H**), 6.38 (d, *J* = 15.8 Hz, 1H, CH = C**H**), 3.79 (s, 3H, OC**H**_3_); ^13^C-NMR (101 MHz, DMSO-*d*_6_) δ 168.41, 161.52, 144.33, 130.52, 127.40, 117.07, 114.93, 55.88, 40.29, 40.08.

## Results and Discussion

Graphene-supported metal NPs based nanocatalysts offer several advantages due to the combined effects of the inherent properties of the components involved. For instance, in the case of HRG-Pd nanocatalysts, the novel catalytic activity of Pd and the large surface area of graphene provide sufficient active sites on the surface of the catalyst leading to the enhancement of the catalytic activity of the resultant composite. However, in many cases, the aggregation of NPs on the surface of the support often diminishes the performance of the material. Besides, the uncontrolled and sporadic growth of NPs on the surface of the support also adversely affects the catalytic potential of supported Pd-based catalysts. To overcome this, herein we have applied non-destructive, π-π interactions-based non-covalent functionalization technique. This was achieved by using 1-amino pyrene as stabilizing ligand, which is a polycyclic aromatic hydrocarbon (PAHs). The PAHs have a strong ability to interact with the 2D flat surface of graphene via π–π interactions due to their unique structures based on fused aromatic rings. The 1-AP functionalized (HRG-Py-Pd) and non-functionalized (HRG-Pd) graphene-palladium nanocomposites and Pd NPs were prepared under facile sonochemical conditions (Khan et al., 2020). Sonochemical preparation is based on high-intensity ultrasound waves which cause acoustic cavitation i.e., the formation, growth, and implosive collapse of bubbles (Xu et al., 2013). The acoustic cavitation generates extreme transient conditions which facilitate the formation of a variety of novel materials, including metallic NPs, under ambient conditions (Prekob et al., 2020). Particularly, high-quality noble metal NPs can be prepared by dissolving nonvolatile precursors in a volatile solvent (usually water or alcohol) (Calcio Gaudino et al., 2018). Such as the *in-situ* deposition of Pd NPs on the surface of HRG, which is performed in this study by using sodium tetrachloropalladate (II) as a nonvolatile precursor and ethanol as a volatile solvent. In this case, ethanol vapors generated by the sonolysis which is caused by high intensity ultrasound waves, act as strong reducing agent, which facilitates the reduction of metal precursor to produce ultrafine Pd NPs. While the ultrasonication produced Pd NPs, the functionalized HRG with 1-AP potentially inhibited the aggregation of NPs and facilitated the homogeneous distribution of Pd NPs on the surface of HRG. In this case, the basal plane of the pyrene ring helps to interact with the surface of HRG through π-π interactions, while, the amine group (NH_2_) of 1-AP promote the nucleation and homogeneous growth of Pd NPs leading to the formation of highly dispersed HRG-Py-Pd nanocatalyst. The as-prepared samples including pristine Pd, HRG-Pd, and HRG-Py-Pd nanocatalysts were applied as a catalyst for the Mizoroki-Heck reaction in the aqueous phase.

### XRD Analysis

**FIGURE 1:**
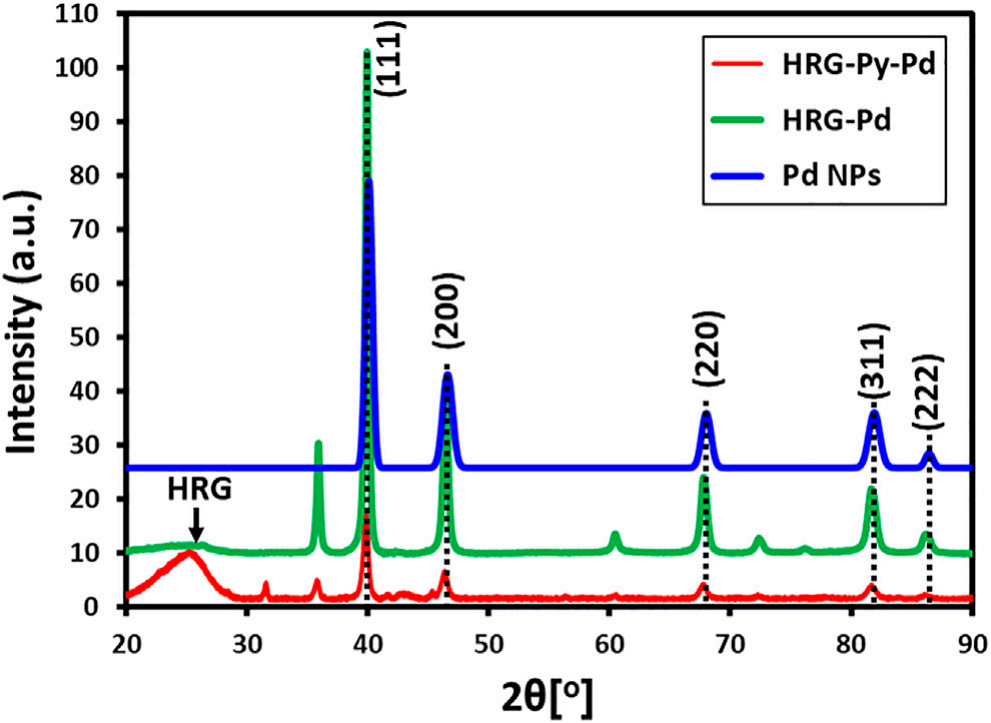
XRD analysis of Pd NPs, HRG-Pd, and HRG-Py-Pd.

Initially, the phase and crystallinity of Pd, HRG-Pd, and HRG-Py-Pd were confirmed by XRD analysis. The XRD diffractograms of these samples are shown in Figure 1. The diffractogram of pristine Pd NPs demonstrates several distinct reflections at 40.02° (111), 46.49° (200), 68.05° (220), 81.74° (311), and 86.24° (222) which are indexed to the face-centered cubic (*fcc*) structure of Pd (ICDD card number PDF#46-1043 (JCPDS: 87-0641), space group: Fm3m (225)) (Khan et al., 2014). On the other hand, the diffractograms of both HRG-Pd and HRG-Py-Pd consist of a broad reflection at 2θ = 22.4° corresponding to the HRG in addition to the reflections belonging to the face-centered cubic (*fcc*) structure of Pd NPs (Khan et al., 2014; Kadam et al., 2020). This indicates the formation of HRG and Pd-based nanocomposites to produce HRG-Pd and HRG-Py-Pd nanocatalysts.

### UV-Visible and FT-IR Analysis

**FIGURE 2:**
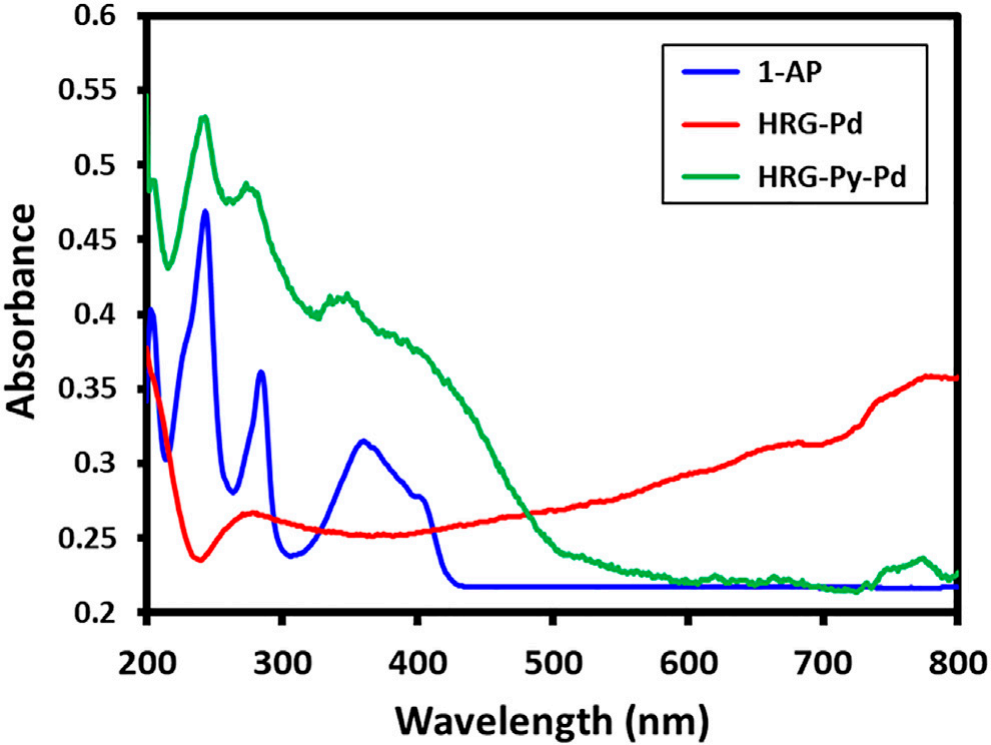
UV-Vis spectrum of pure 1-amino pyrene, HRG-Pd, and HRG-Py-Pd.

**FIGURE 3:**
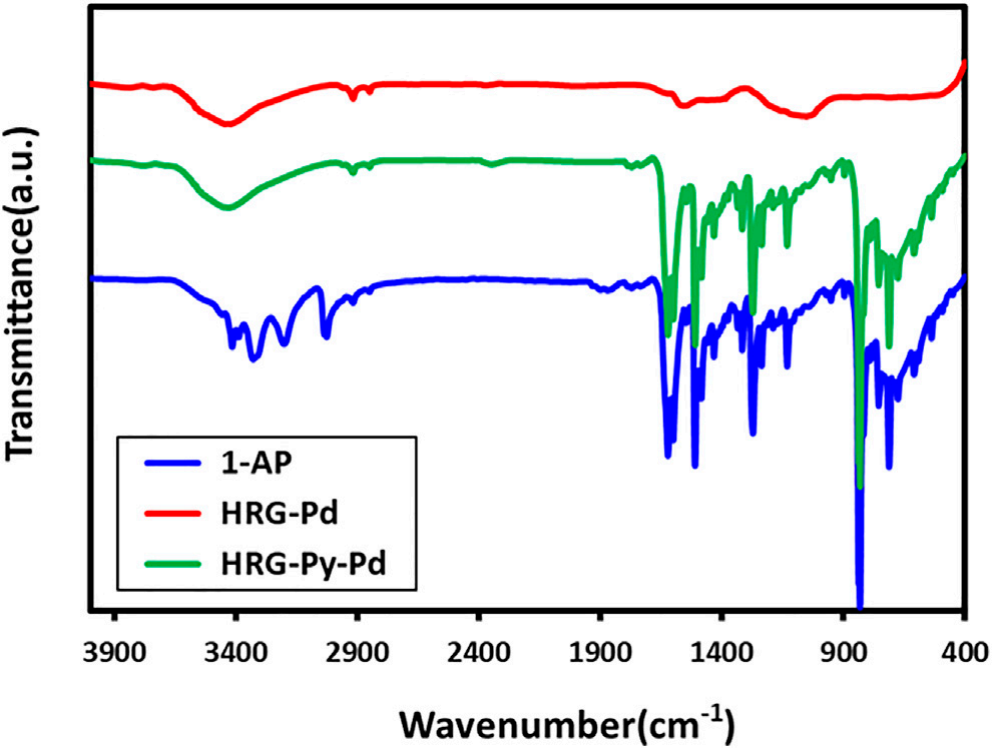
FT-IR spectrum of pure 1-amino pyrene, HRG-Pd, and HRG-Py-Pd.

The successful functionalization of HRG with1-AP in HRG-Py-Pd is confirmed by both UV-Vis and FT-IR analyses. For this purpose, the UV spectra of 1-AP, HRG-Pd, and HRG-Py-Pd are compared as shown in Figure 2. The UV spectrum of 1-AP exhibits three characteristic bands at ∼242, ∼285, and 360 nm (Figure 2) which are also present in the UV spectrum of HRG-Py-Pd (Figure 2). Apart from these (1-AP) bands, the HRG-Py-Pd also consists of a characteristic absorption band of HRG, which typically appears at ∼270 nm, however, in this case, it is not clearly visible due to the coexistence of the 1-AP absorption band in a similar region (∼285 nm). Notably, the UV spectrum of HRG-Pd only exhibits an absorption band at ∼270 nm belonging to HRG, thus the absence of the absorption bands of 1-AP in the UV spectrum of HRG-Pd is also an indication of the successful functionalization of the surface of HRG with 1-AP in HRG-Py-Pd. Similarly, FT-IR analyses of these samples also confirmed the non-covalent functionalization of HRG by 1-AP in HRG-Py-Pd, as revealed by the comparison of the IR spectra of 1-AP, HRG-Pd, HRG-Py-Pd (cf. Figure 3). The IR spectrum of 1-AP exhibits several characteristic peaks, and most of these peaks also appeared in the IR spectrum of HRG-Py-Pd. This clearly indicated the presence of 1-AP on the surface of HRG in HRG-Py-PD. For example, the IR peaks of the fingerprint region of 1-AP between 800 and 1,700 cm^−1^ are also present in the IR spectrum of HRG-Py-Pd, whereas, these characteristic peaks are absent in the IR spectrum of HRG-Pd which points towards the absence of 1-AP in this sample.

### Transmission Electron Microscopy and EDX Analysis

**FIGURE 4:**
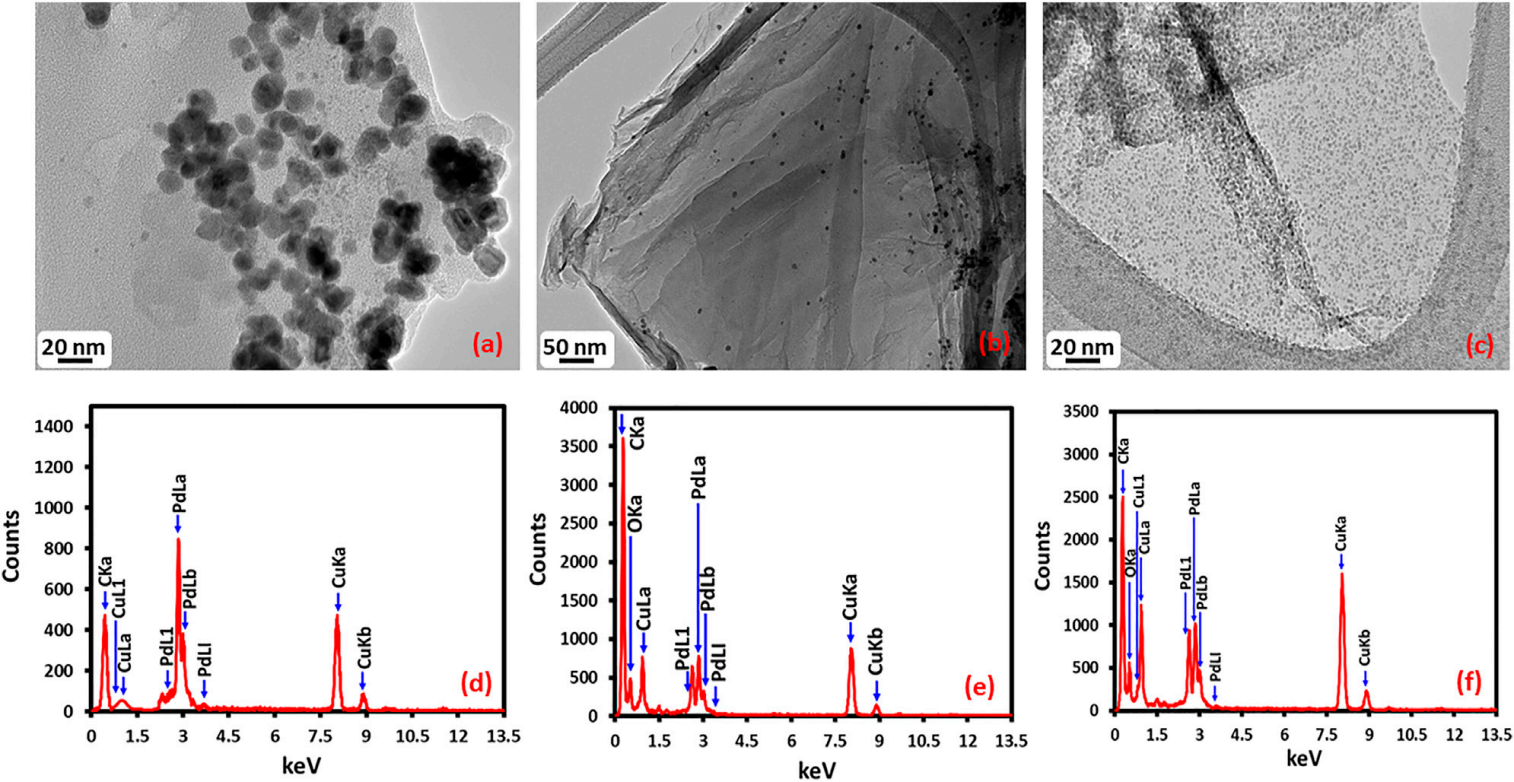
HR-TEM images of **(A)** Pd NPs, **(B)** HRG-Pd, **(C)** HRG-Py-Pd and EDX spectra of **(D)** Pd NPs, **(E)** HRG-Pd, **(F)** HRG-Py-Pd.

**FIGURE 5:**
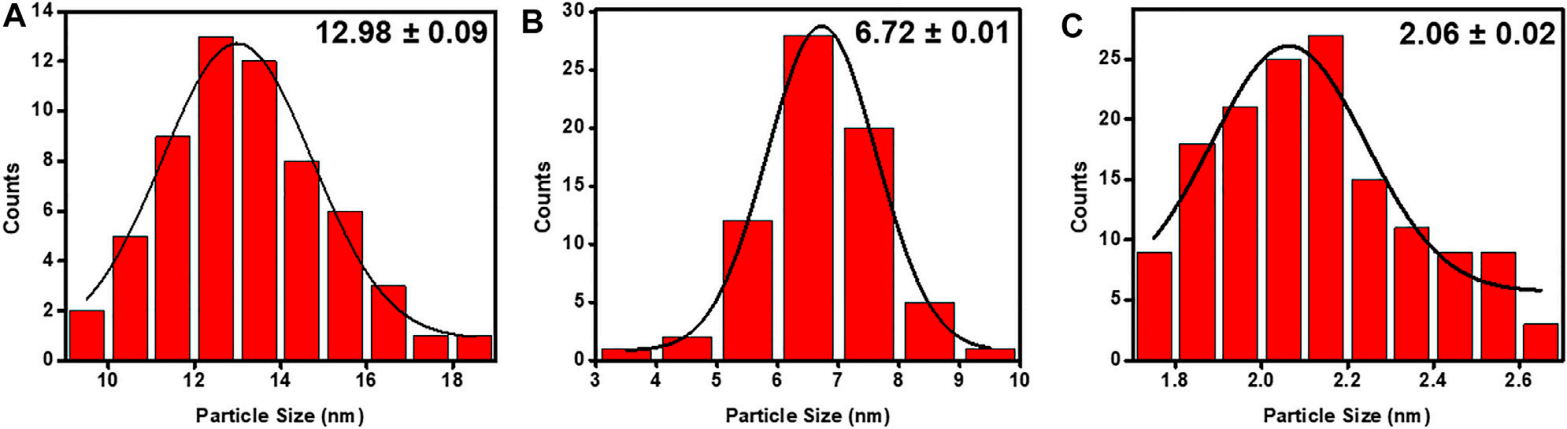
Particle Size distribution graph of **(A)** Pd NPs, **(B)** HRG-Pd, and **(C)** HRG-Py-Pd.

Morphology and size of the as-prepared Pd NPs, HRG-Pd, and HRG-Py-Pd were determined using transmission electron microscopy (TEM) (Figure 4). The overview image in Figure 4A exhibits the formation of spherically shaped, almost homogeneous size Pd NPs with a size distribution of ∼20–25 nm. The elemental composition of the as-prepared NPs as determined by using EDX confirmed the presence of only Pd. The morphology and the structure of Pd NPs on the surface of HRG in HRG-Pd is shown in the overview image in Figure 4B which indicates the existence of small and spherical shape Pd NPs on the surface of HRG. Whereas, the results of the elemental composition of the as-prepared HRG-Pd nanocatalyst determined by EDX (Figure 4E), revealed the presence of both carbon and Pd NPs. Besides, the size and morphology of Pd NPs on the surface of HRG in HRG-Py-Pd were also determined using transmission electron microscopy (TEM). The TEM micrograph of HRG-Py-Pd is presented in Figure 4C. It shows the presence of homogeneously dispersed ultrafine small size Pd NPs on the surface of HRG. Unlike in HRG-Pd which exhibited relatively larger size (10-20 nm), highly aggregated Pd NPs, the HRG-Py-Pd demonstrate the presence of densely distributed, spherical-shaped, smaller size (∼2–4 nm) Pd NPs due to the presence of 1-AP. The presence of Pd NPs onto HRG-Py-Pd is also confirmed in the EDX spectrum of the functionalized nanocatalyst in Figure 4F. Notably, the Pd to C ratio is relatively higher in HRG-Py-Pd when compare with the Pd:C in HRG-Pd nanocatalyst (Figure 4). Additionally, the particle size distribution graph was assessed by using ImageJ software (Figures 4A–C), the particle size distribution graph of the Pd NPs (Figure 5A) displays the average particle size 12.98 ± 0.09 nm, the particle size distribution graph of the HRG-Pd (Figure 5B) displays the average particle size 6.72 ± 0.01 nm and the particle size distribution graph of the HRG-Py-Pd (Figure 5C) displays the average particle size 2.06 ± 0.02 nm. This indicates that 1-AP effectively promotes the nucleation and uniform growth of Pd NPs and also inhibits the aggregation of HRG nanosheets may enhance the surface area of the material, while the apparent dense distribution of Pd on the surface of HRG offers increased catalytic active sites.

**SCHME 1:**
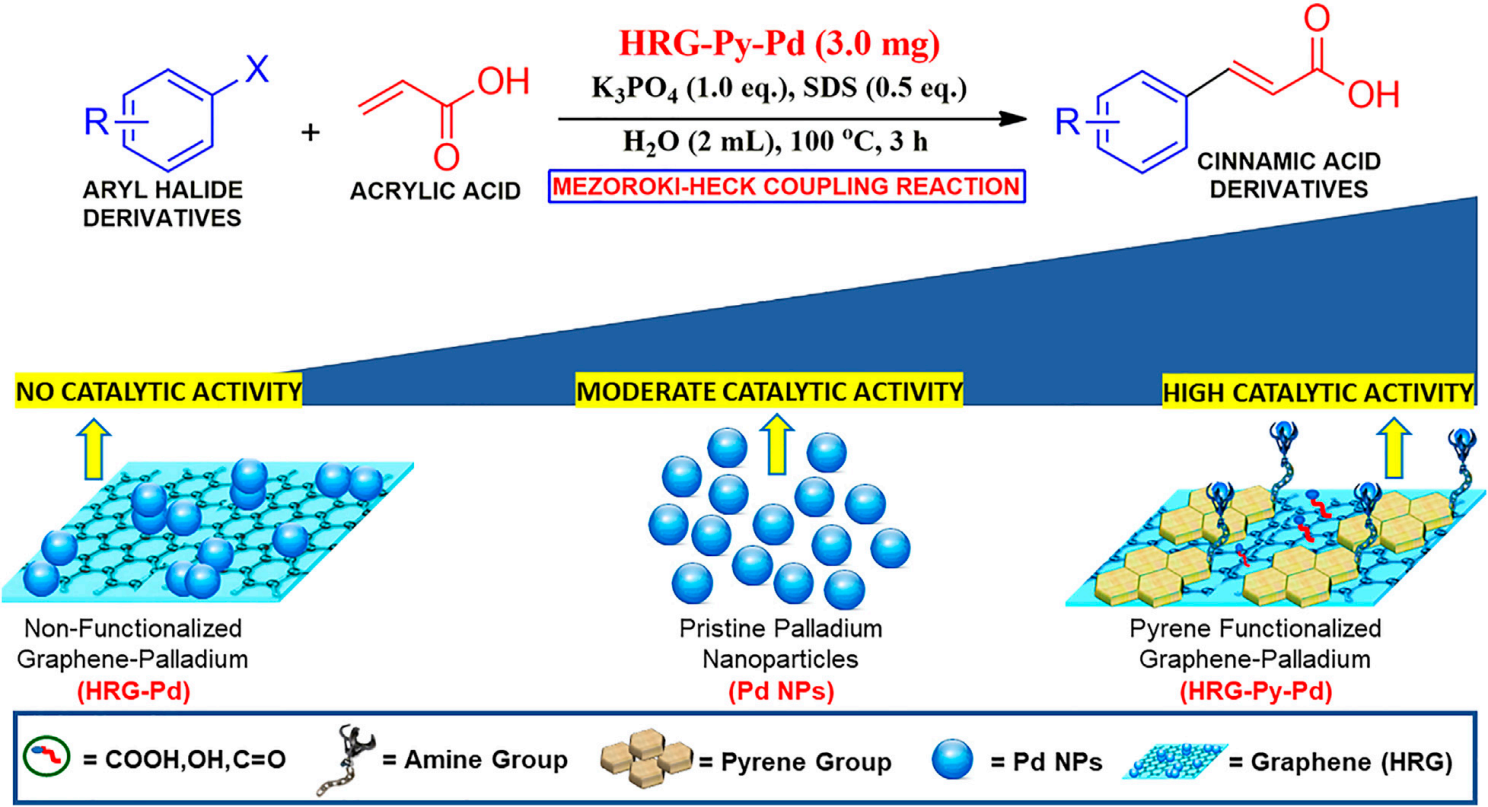
Schematic representation of the HRG-Py-Pd catalyzed Mizoroki-Heck coupling reaction in an aqueous medium.

#### Catalytic Application

Water dispersible Pd-based heterogeneous catalysts are highly required for various catalytic transformations including the Mizoroki-Heck reaction in the aqueous phase. Particularly, Pd NPs immobilized on various solid supports including carbonaceous materials, silica, and metal oxide NPs have demonstrated excellent catalytic activity for these types of coupling reactions. Therefore, heterogeneous catalysts based on Pd have attracted significant attention for the Mizoroki-Heck reactions under aqueous conditions. However, in many cases, the catalytic efficiency of Pd-based nanocatalysts is seriously hindered due to the aggregation of nanoparticles on the surface of the support. This is typically avoided through the functionalization of nanocatalyst with effective ligands. For instance, in our previous study, we have demonstrated the enhancement of the catalytic activity of HRG-Py-Pd nanocatalyst in the Suzuki–Miyaura coupling reactions. Stabilization of the surface of HRG with 1-AP has enhanced the dispersibility and uniformity of nanocatalyst on the support surface and solubility in water. This improved the efficiency of the catalyst compared to its non-functionalized counterpart (HRG-Pd). 1-AP with its active amine group offered efficient binding sites for the growth of well-separated, densely distributed Pd NPs on the surface of HRG, which increased the surface area of the resulting nanocatalyst. In this study, we investigated the catalytic activity of both functionalized (HRG-Py-Pd) and non-functionalized HRG-Pd for the Mizoroki-Heck reactions under aqueous conditions. Besides, the catalytic activity of these nanocatalysts is also compared with the pristine Pd NPs which were prepared using the ultrasonication method. In order to examine the catalytic efficiency of our freshly prepared nanocatalysts (Pd NPs, HRG-Pd, and HRG-Py-Pd) for the Mizoroki-Heck cross-coupling reaction, 4-Bromoanisol (**1a**) and acrylic acid (**2**) have been chosen as model substrate and the reaction was performed in aqueous medium as shown in Scheme 2.

**SCHME 2:**
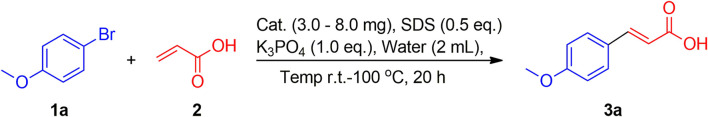
Mizoroki-Heck reaction (MH) of 4-bromoanisol and acrylic acid in aqueous solution using newly prepared nanocatalysts (Pd-NPs, HRG-Pd, and HRG-Py-Pd).

**SCHEME 3:**
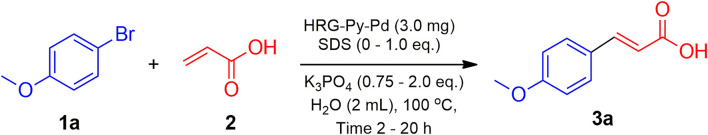
Mizoroki-Heck reaction of 4-bromoanisol and acrylic acid in aqueous medium using Pd-NPs and HRG-Py-Pd nanocatalysts in aqueous medium at 100°C.

The Mizoroki-Heck cross-coupling reaction of 4-bromoanisol **1a** (0.5 mmol) and acrylic acid **2** (0.5 mmol) as model substrate was carried out using freshly prepared catalyst Pd-NPs, HRG-Pd, and HRG-Py-Pd (3.0–8.0 mg) in the presence of K_3_PO_4_ (1.0 mmol) and SDS (0.25 mmol) in water (2 ml) at different temperatures such as room temperature (r.t.), 90°C, and 100 ^o^C for 20 h (cf. Scheme 3). The results are summarized in Table 1. Initially, catalyst Pd-NPs, HRG-Pd, and HRG-Py-Pd (5.3 mg) were used in order to carry out the MH-reactions using K_3_PO_4_ (1.0 mmol) and SDS (0.25 mmol) at r.t. as well as at 90°C for 20 h, but unfortunately, no MH product formation was observed (Table 1, entries 1, 2, 6, 7, 11, 12). The reaction temperature was then elevated to 100°C, keeping other parameters unchanged, catalysts Pd-NPs and HRG-Py-Pd performed extremely well, producing MH coupling products 4-Methoxycinnamic acid (**3a**) with 92 and 84% conversion respectively (Table 1, entries 4, 14) while catalyst HRG-Pd remained inactive (Table 1, entry 8). To optimize catalyst loading, the MH reactions were further performed using 3.0–8.0 mg of each catalyst (Pd-NPs, HRG-Pd, and HRG-Py-Pd). It is observed that, the catalyst Pd-NPs produced 40% (3 mg) and 74% (8 mg) conversion (Table 1, entries 3 and 5) whereas catalyst HRG-Py-Pd furnished 93% (3 mg) and 71% (8 mg) conversion (Table 1, entries 13 and 15) respectively. Catalyst HRG-Pd was again found to be inactive even at higher temperatures and high catalyst loading (Table 1, entries 8 and 10). Interestingly catalyst HRG-Py-Pd (3.0 mg) performed excellently as compared to Pd-NPs (3 mg) at expense of lower catalyst usage (Table 1, entries 13, 3). Upon increasing the catalyst loading to 8 mg, yields were not improved for both the catalyst Pd-NPs and HRG-Py-Pd, in fact, conversion significantly dropped to 74 and 71% (Table 1 entries 5 and15) which could be because of the aggregation of particles (particle-particle interactions) which tend to decrease the exposed surface area of the catalyst active sites. Therefore, our preliminary investigation revealed that 5.3 mg of catalyst Pd-NPs and 3.0 mg of catalyst HRG-Py-Pd are the best choice for catalyzing MH reaction of 4-bromoanisol and acrylic acid in water as a green medium at 100°C. Notably, non-functionalized HRG-Pd proved to be an inactive catalyst that did not produce any conversion even after a long reaction time, higher temperature, and using a high amount of catalyst. Although pristine Pd-NPs were active for the coupling reaction of 4-bromoanisol and acrylic acid in an aqueous medium, the inactivity of HRG-Pd under similar reaction conditions can be attributed to the severe aggregation of Pd-NPs on the surface of HRG which is also established by the HRTEM results. Aggregated NPs not only decreased the number of active sites but also affected the dispersibility of nanocatalysts in the aqueous medium, which led to the inactivity of the HRG-Pd.

**TABLE 1:** Mizoroki-Heck reaction (MH), 4-bromoanisol and acrylic acid as a model substrate. Catalyst screening.

Sl. No	Catalyst	Cat. Wt. (mg)	K3PO4 (eq.)	SDS (Eq.)	Temp (oC)	HPLC Conversion
1	Pd-NPs	5.3	2	0.5	R.T.	—
2		5.3	2	0.5	90°C	traces
3		3.0	2	0.5	100°C	40%
4		5.3	2	0.5	100°C	92%
5		8.0	2	0.5	100°C	74%
6	HRG-Pd	5.3	2	0.5	R.T.	—
7		5.3	2	0.5	90°C	—
8		3.0	2	0.5	100°C	—
9		5.3	2	0.5	100°C	—
10		8.0	2	0.5	100°C	—
11	HRG-Py-Pd	5.3	2	0.5	R.T.	—
12		5.3	2	0.5	90°C	traces
13		3.0	2	0.5	100°C	93%
14		5.3	2	0.5	100°C	84%
15		8.0	2	0.5	100°C	71%

Reaction condition: 4-bromoanisol (0.5 mmol) and acrylic acid (0.5 mmol), catalyst (3.0–8.0 mg), K3PO4 (1.0 mmol), SDS (0.25 mmol) and water (2 ml) at R.T.—100°C, time 20 h.

After examining the efficiency of all the as-prepared nanocatalysts at different catalyst loading, the effect of reagents (SDS, K_3_PO_4_), solvent, and reaction time were evaluated on the same model MH reaction, using the best performing catalyst mass loading condition i.e. Pd-NPs (5.3 mg) and HRG-Py-Pd (3.0 mg), respectively, as both the catalyst demonstrated best results with small amount of catalyst loading and the outputs are shown in Table 2. When reactions were performed in the absence of SDS using both the catalysts Pd-NPs (5.3 mg) and HRG-Py-Pd (3.0 mg), no conversion was observed (Table 2, entries 1 and 5). The MH reaction was further carried out by using 0.25 eq., 0.5 eq., and 1.0 eq. of SDS without changing other parameters, and 0.5 eq. of SDS was found to be the best choice for this catalytic reaction as it produced 92 and 93% conversion respectively (Table 2, entries 3 and 7). This is in accordance with the literature, where the role of SDS as a stabilizing agent in Pd catalyzed coupling reactions is well established. For example, in the absence of SDS either the reaction does not occur or the yield of the reaction is sharply decreased (Sherwood, James, et al., 2019). After optimizing the amount of SDS (0.5 eq.), we focused on the effects of K_3_PO_4_ under the optimized parameters (catalyst 5.3 mg of Pd-NPs and 3.0 mg HRG-Py-Pd mg, 0.5 eq. of SDS in water 2 ml at 100°C for 20 h). Therefore, MH reactions were carried out using 0.75 eq. as well as1.0 eq. of K_3_PO_4_ without altering the other optimized parameters, and the conversions sharply increased from 68 to 98% (Table 2, entries 12 and 13) in the case of catalyst HRG-Py-Pd, while significant improvement in conversion was achieved 20–37% (Table 2, entries 9 and 10) in case of catalyst Pd-NPs at a much slower pace as compared to catalyst HRG-Py-Pd. Further, we increased the amount of K_3_PO_4_ to 1.5 eq. in order to achieve further improvement in the reaction using both the catalysts under the identical conditions, and this time yields of the reaction doubled from 37 to 78% (Table 2, entries 10 and 11) while using Pd-NPs; however, in case of HRG-Py-Pd, virtually no effect on the conversion (97%) were observed it almost remained same (Table 2, entries 14). Finally, reaction time optimization has been done using both the catalyst under the individual optimized reaction parameters. The MH reaction was performed using Pd-NPs (5.3 mg) as the catalyst and other optimized parameters such as K_3_PO_4_ (2.0 eq), SDS (0.5 eq.) in water (2.0 ml) at 100°C for 2, 3, and 8 h and the respective yields (20, 36, and 62%) were observed (Table 2, entries 15–17). These findings tell us that the yield of the product is continuously increasing with time at a slower pace while using Pd-NPs as the catalyst. As we know from the previous observation catalyst Pd-NPs took a longer time (20 h) to obtain 92% conversion (Table 1, entry 4). On the contrary, using HRG-Py-Pd as a catalyst under the optimized condition [K_3_PO_4_ (1.0 eq.), SDS (0.5 eq.) in water (2.0 ml) at 100°C], MH reactions were carried out for 2, 3, and 8 h. The results were surprising and fascinating as 99% conversion was achieved just within 3 h (Table 2, entry 19). However, very good conversion (90%) was noticed just in 2 h while 98% were observed after 8 h (Table 2, entries 18 and 20). From Table 2 findings, the time of reaction has a diverse effect on the conversion of the reaction when different catalysts were used. For example, in the case of catalyst Pd-NPs, a longer reaction time was required as the conversion of product increases slowly with time, whereas, functionalized catalyst HRG-Py-Pd completed the conversion in a very short time compared to Pd-NPs. Notably, in just 3 hours with 3.0 mg of catalyst HRG-Py-Pd, almost complete conversion (99%, Table 2, entry 19). Additionally, the effect of solvent was evaluated using DMF as a solvent instead of water. It is noteworthy to mention that the reaction did not occur at all when water is replaced with DMF (Table 2, entry 21 and 22), in the case of both the catalysts. This can be attributed to the low dispersibility of catalysts in solvents other than water.

**TABLE 2:** Mizoroki-Heck reaction of 4-bromoanisol (1a) and acrylic acid (2) in aqueous solution using best catalyst HRG-Py-Pd; Optimization for K3PO4, SDS, and reaction time.

Sl. No	Catalyst	Cat. Wt. (mg)	K3PO4 (Eq.)	SDS (Eq.)	Time (h)	HPLC Conversion
SDS optimization						
1	Pd-NPs	5.3	2	—	20	—
2		5.3	2	0.25	20	19%
3		5.3	2	0.5	20	92%
4		5.3	2	1.0	20	88%
5	HRG-Py-Pd	3.0	2	—	20	—
6		3.0	2	0.25	20	24%
7		3.0	2	0.5	20	93%
8		3.0	2	1.0	20	92%
K3PO4 Optimization						
9	Pd-NPs	5.3	0.75	0.5	20	20%
10		5.3	1.0	0.5	20	37%
11		5.3	1.5	0.5	20	78%
12	HRG-Py-Pd	3.0	0.75	0.5	20	68%
13		3.0	1.0	0.5	20	98%
14		3.0	1.5	0.5	20	97%
Reaction Time Optimization						
15	Pd-NPs	5.3	2.0	0.5	2	20%
16		5.3	2.0	0.5	3	36%
17		5.3	2.0	0.5	8	62%
18	HRG-Py-Pd	3.0	1.0	0.5	2	90%
19		3.0	1.0	0.5	3	99%
20		3.0	1.0	0.5	8	98%
Different Solvent System (DMF)						
21	Pd-NPs	5.3	2.0	0.5	20	-
22	HRG-Py-Pd	3.0	1.0	0.5	20	-

Reaction condition: 4-bromoanisol 1a (0.5 mmol) and acrylic acid 2 (0.5 mmol), catalyst used Pd-NPs, 5.3 mg and HRG-Py-Pd 3.0, water (2 ml) at 100°C, time 2–20 h.

**SCHEME 4:**
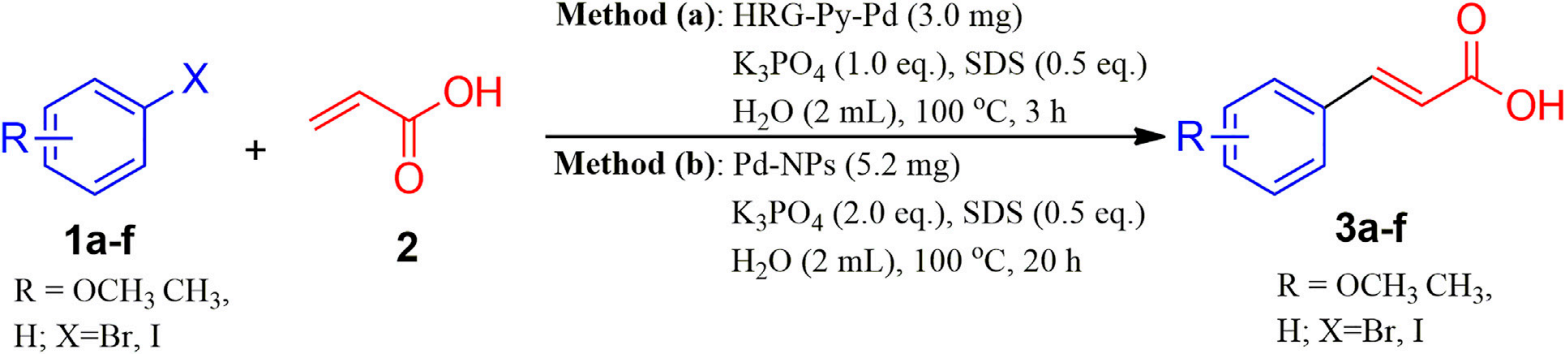
Mizoroki-Heck reaction substrate scope under optimized reaction conditions using HRG-Py-Pd and Pd-NPs nanocatalysts.

To further explore the scope of functionalized nanocatalysts HRG-Py-Pd and non-functionalized Pd-NPs on different substrates in cross-coupling reaction, a series of aryl halides with diverse organic functional groups were applied as substrates in the same model reaction under the above-optimized reaction condition. For instance, in the case of Pd-NPs, 5.3 mg of catalyst, 2.0 Eq. of K_3_PO_4_, 0.5 Eq. of SDS, 2 ml of H_2_O, and 20 h of reaction time were applied, whereas, for HRG-Py-Pd, the amount of catalyst and the reaction time was reduced to 3 mg and 3 h respectively (cf. Scheme 4). The results of these reactions are summarized in Table 3, which indicate that the as-prepared nanocatalysts have efficiently facilitated the coupling reactions of diverse aryl halides including phenyl iodide, phenyl bromide, etc., with acrylic acid under optimized conditions. These diverse aryl halides with substituents at different positions have resulted in the formation of corresponding cinnamic acid derivatives in very good yield ranging from 83 to 92% in the case of Pd-NPs (5.3 mg) with 20 h reaction time, whereas, HRG-Py-Pd (3.0 mg) has delivered excellent yield between 87 and 99% for the same reactions (Table 3, entries 1–6). Notably, in both cases, the substrates with functional groups at ortho positions have yielded a lower amount of product as compared to meta and para substitution (Table 3, entry 3), which can be attributed to the higher steric hindrance caused by the substituents at ortho position positions. However, iodo-benzene and iodo-toluene furnished maximum yields in the case of both the catalysts (Table 3, entries 4 and 5). Both the catalysts were unable to produce MH products when 2-bromopyridin was used as substrate (Table 3, entry 7).

**TABLE 3:** Mizoroki-Heck reaction catalyzed by HRG-Py-Pd and Pd-NPs nanocatalysts, Substrate scope.

Sl. No	Aryl Halides	1a-g	3a-f	Yielda,c HRG-Py-Pd	Yieldb,cPd-NPs
1	4-bromoanisol	1a	3a	98% (99%)d	91% (93%)d
2	3-bromoanisol	1b	3b	94%	88%
3	2-bromoanisol	1c	3c	87%	83%
4	4-Iodotoluene	1d	3d	98%	92%
5	4-Iodobenzene	1e	3e	98%	90%
6	4-bromobenzene	1f		96%	87%
7	2-bromopyridine	1g	3f	—	—

Aryl halides (0.5 mmol), Acrylic acid (0.5 mmol); a Catalyst HRG-Py-Pd (3.0 mg), K3PO4 (1.0 eq.), SDS (0.5 eq.) in H2O (2 ml), 100°C, 2 h; b Catalyst Pd-NPs (5.3 mg), K3PO4 (2.0 eq.), SDS (0.5 eq.) in H2O (2 ml), 100°C, 20 h; c Isolated yields; d HPLC, conversion.

Additionally, the efficacy of the HRG-Py-Pd was determined by comparing the data published in the literature with the results of Mizoroki-Heck coupling reaction of 4-bromoanisol and acrylic acid under similar reaction conditions obtained with a variety of supported Pd NPs based heterogeneous catalysts. Conversion values obtained from the literature are listed in Table 4, which indicate that the functionalized HRG-Py-Pd nanocatalyst evaluated in this study has delivered a much higher catalytic activity and is relatively superior to other catalyst systems used for this reaction. Therefore, the non-covalent pyrene functionalized HRG-Py-Pd nanocatalyst applied in this study is an effective approach in terms of the use of efficient support (HRG, consisting of high surface area and enormous active sites), smart design, ease of deployment of catalyst, high dispersibility in water, versatility, i.e., compatibility with a wide range of systems, and high conversion.

**TABLE 4:** Comparison of the results obtained with the HRG-Py-Pd nanocatalyst for the Mizoroki-Heck reaction of 4-bromoanisol and acrylic acid in an aqueous solution with previously reported results in the literature.

S. No	Catalyst System	Base	Solvent System	Temp. (°C)	Conversion (%)	References
1	HRG-Py-Pd	K3PO4	H2O	100	99	This study
2	Pd@SP-CMP	K2CO3	DMF	80	95	Ju et al. (2019)
3	MPCS-TI/Pd	Et3N	DMF/H2O	110	91	Rezaei 2015
4	Fe3O4@PCA/Pd (0)-b-PEG	K2CO3	H2O	90	91	Tabatabaei Rezaei et al. (2017)
5	PANI-Pd	K2CO3	DMA	120	85	Patel et al. (2015)
6	Pd-CS@SiO2	K2CO3	DMF	110	85	Jadhav et al. (2015)
7	Pd(OAc)2	ET3N	H2O	100	79	Patil et al. (2020)

To test the stability and reusability of HRG-Py-Pd, the coupling of 4-bromoanisol **1a** (3 mmol) and acrylic acid **2** (3 mmol) was used as a model reaction which generated the highest yield (99%). The reaction was performed for 3 h using 18 mg of HRG-Py-Pd, water (12 ml) at 100°C. The stability of the catalyst was tested up to five reactions (5 cycles) (Supplementary Figure 37A,B). After every reaction, the catalyst was recovered via centrifugation, washed three times with DI water, dried in an oven, and reused. Fresh catalyst in the first reaction yielded 99% conversion, while in the successive reactions, the catalyst activity reduced negligibly up to ∼6–8% as shown in Supplementary Figure 37B in the supplementary information. Even after five cycles, the structure of HRG-Py-Pd catalyst almost remained intact which is confirmed by XRD analysis Supplementary Figure 37A.

## Conclusion

Herein, a non-covalently functionalized HRG, decorated with Pd nanoparticles (HRG-Py-Pd) based nanocomposite is prepared, characterized, and applied as a nanocatalyst for the Mizoroki-Heck reaction in an aqueous medium. Initially, the catalytic efficacy of HRG-Py-Pd is tested for the conversion of 4-bromoanisol and acrylic acid in water. The activity of HRG-Py-Pd is also compared with non-functionalized HRG-Pd and pristine Pd NPs based nanocatalysts. Among all the catalysts, the HRG-Py-Pd has delivered the best results and yielded an almost complete conversion of 4-bromoanisol and acrylic acid to corresponding cinnamic acid (99%) in water. The reaction occurred in a short time (3 h) and just required a relatively small amount of catalyst (3 mg). On the other hand, with the same amount of catalysts (3 mg), the pristine Pd NPs just yielded a meager 40% conversion even after 20 h, while the non-functionalized HRG-Pd did not deliver any results and proved to be ineffective for the same reaction (cf. Table 5). The superior catalytic activity of HRG-Py-Pd is ascribed to the smart design of nanocatalyst consisting of pyrene as the functionalizing ligand because; 1) it significantly increased the physical stability and dispersibility of the catalyst in water, and 2) it provides nucleation sites for the homogenous and uniform growth of Pd nanoparticles. Besides, a variety of aryl halides are also investigated for the Suzuki-Miyaura C-C bond formation reaction and the use of HRG-Py-Pd also resulted in high conversion. Therefore, the HRG-Py-Pd due to its high dispersibility delivered excellent catalytic activity for the coupling reactions in water, and thus offers a new route for the development of similar catalysts with other metal nanoparticles for a range of other catalytic transformations.

**TABLE 5:** Comparison of the reaction yield of Mizoroki-Heck reaction catalyzed by pristine Pd-NPs, HRG-Pd, and functionalized HRG-Py-Pd nanocatalysts using the same amount of catalysts under similar reaction conditions.

Sl. No	Catalyst	Cat. Wt. (mg)	K3PO4 (eq.)	SDS (Eq.)	Time (h)	Temp (oC)	HPLC Conversion
1	Pd-NPs	3.0	2	0.5	20	100	40%
2	HRG-Pd	3.0	2	0.5	20	100	—
3	HRG-Py-Pd	3.0	2	0.5	3	100	99%

## Data Availability

The raw data supporting the conclusions of this article will be made available by the authors, without undue reservation. Additional information on HPLC chromatograms and NMR spectra is provided in the supplementary material (Supplementary Figures S1–S36).
