# Supplementary figures and images for: Pyrene Functionalized Highly Reduced Graphene Oxide-palladium Nanocomposite: A Novel Catalyst for the Mizoroki-Heck Reaction in Water
Source: Front Chem. 2022 Apr 29;10:872366. doi: 10.3389/fchem.2022.872366 (PMC9101052; doi:10.3389/fchem.2022.872366)

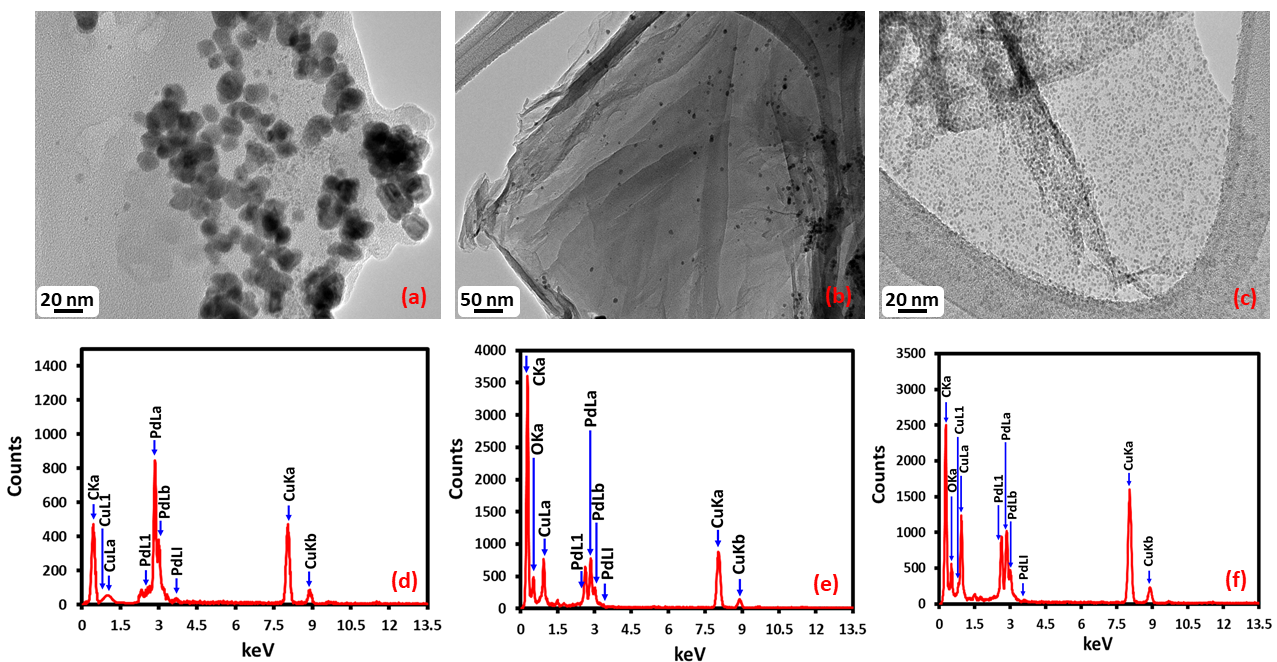

Supplement: Supplementary file 1 [file Figure5.TIF]

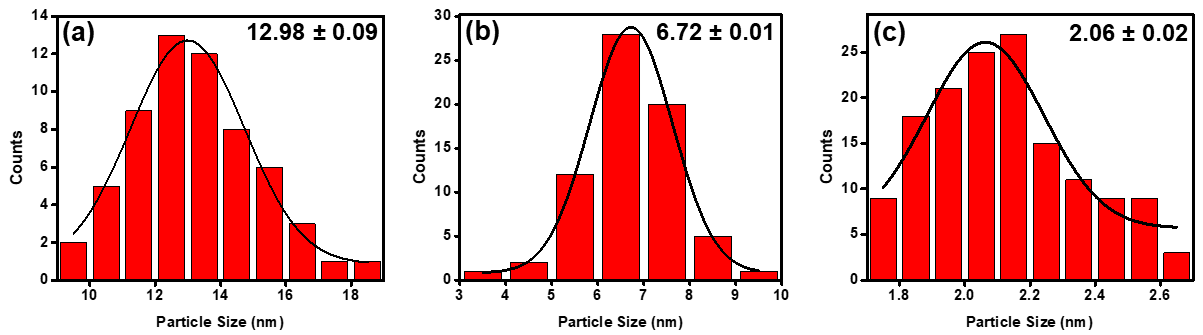

Supplement: Supplementary file 2 [file Figure6.TIF]

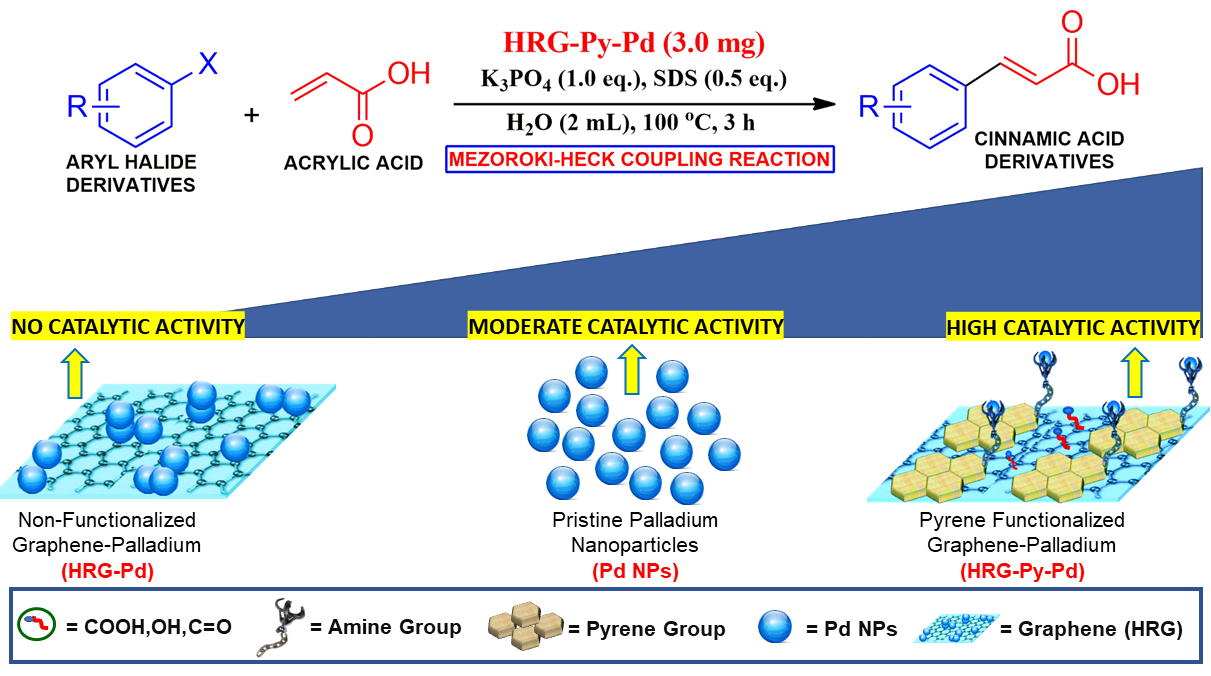

Supplement: Supplementary file 3 [file Figure1.TIF]

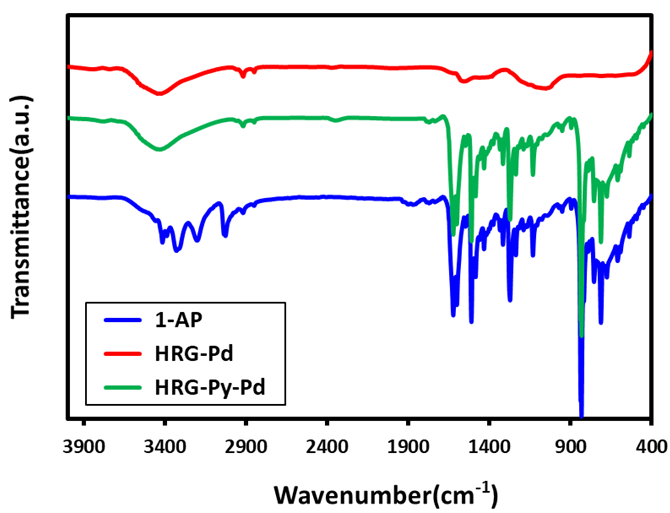

Supplement: Supplementary file 4 [file Figure4.TIF]

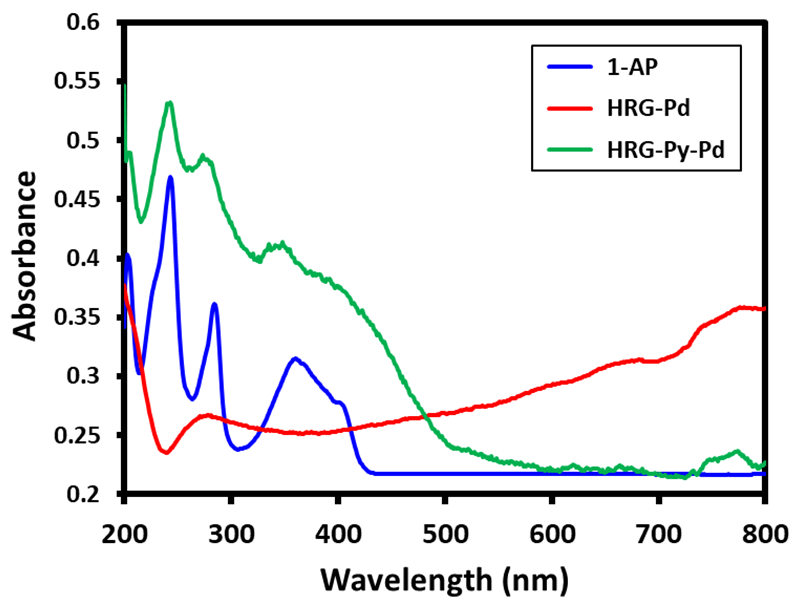

Supplement: Supplementary file 6 [file Figure3.TIF]

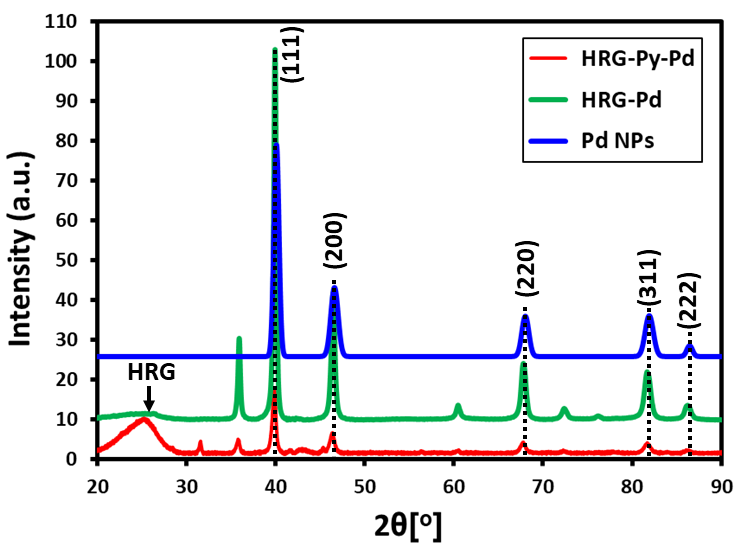

Supplement: Supplementary file 8 [file Figure2.TIF]
