# Supplementary material for: Pyrene Functionalized Highly Reduced Graphene Oxide-palladium Nanocomposite: A Novel Catalyst for the Mizoroki-Heck Reaction in Water
Source: Front Chem. 2022 Apr 29;10:872366. doi: 10.3389/fchem.2022.872366 (PMC9101052; doi:10.3389/fchem.2022.872366)
Supplement: Supplementary file 10 [file DataSheet1.docx]

Supplementary Material

Pyrene Functionalized Highly Reduced Graphene Oxide-Palladium Nanocomposite: A Novel Catalyst for the Mizoroki-Heck reaction in Water

Mujeeb Khan^1,^*, Mohammed Rafi Shaik^1^, Syed Farooq Adil^1^, Mohammad Shahidul Islam^1,^*, Mufsir Kuniyil^1^, Merajuddin Khan^1^, Mohammad Rafe Hatshan^1^, Riyadh H. Alshammari^1^, Mohammed Rafiq H. Siddiqui^1^, Muhammad Nawaz Tahir^2^

^1^Department of Chemistry, College of Science, King Saud University, P.O. 2455, Riyadh 11451, Saudi Arabia

^2^Chemistry Department, King Fahd University of Petroleum & Minerals, Dhahran 31261, Saudi Arabia

^*^ **Correspondence:**Mujeeb Khan
[kmujeeb@ksu.edu.sa](mailto:kmujeeb@ksu.edu.sa)

Mohammad Shahidul Islam

[mislam@ksu.edu.sa](mailto:mislam@ksu.edu.sa)

# HPLC analysis


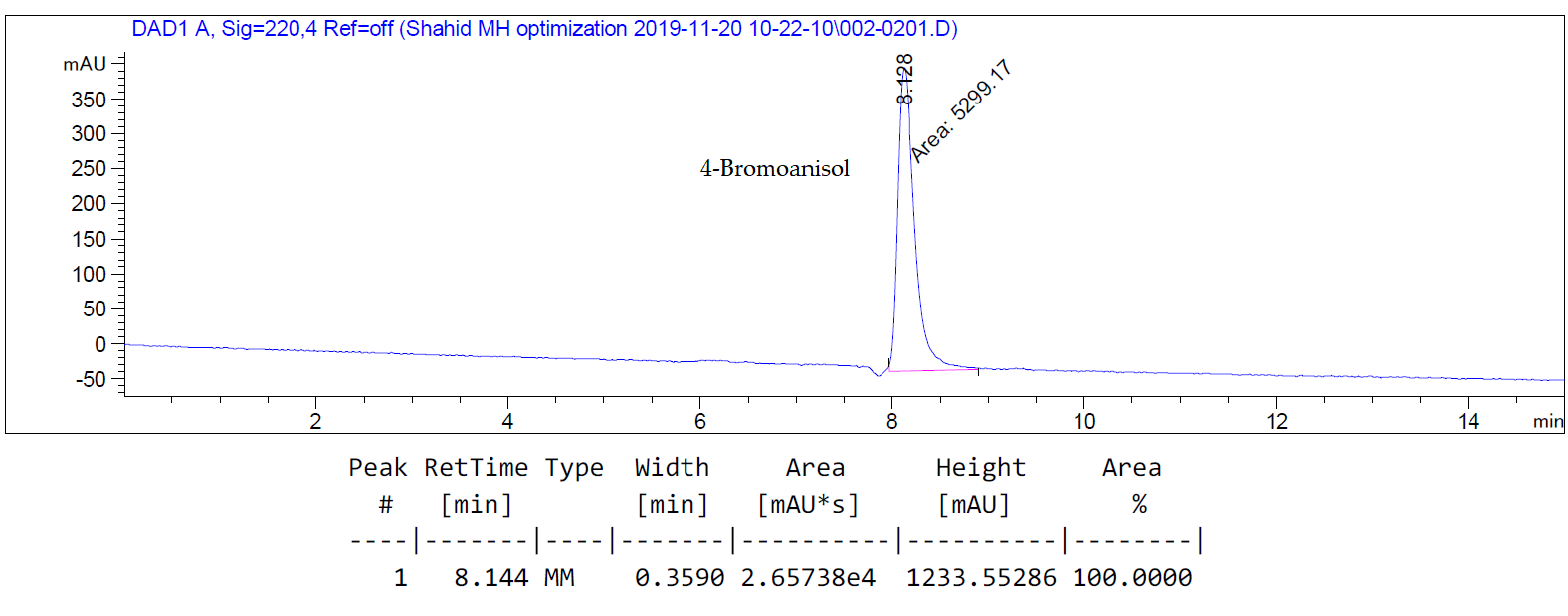


## Supplementary Figure 1. Control sample 4-bromoanisol.


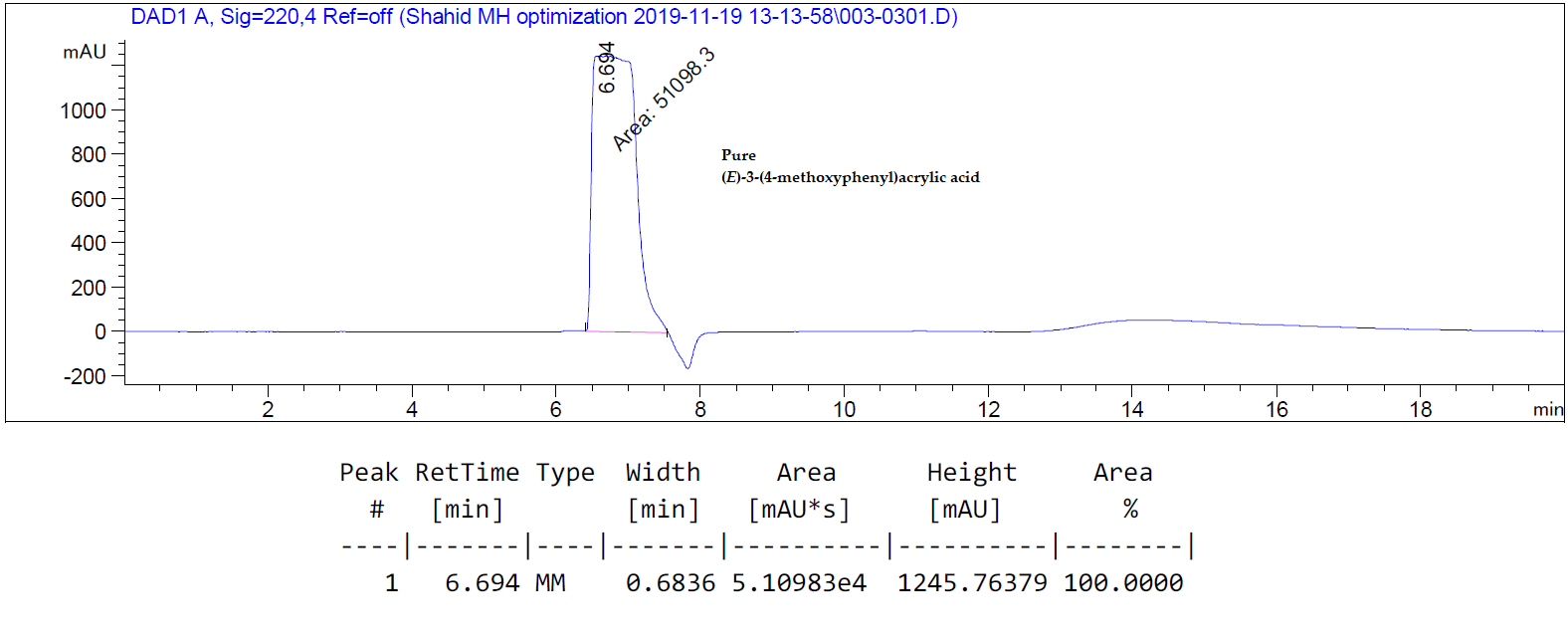


## Supplementary Figure 2. Control sample (*E*)-3-(4-methoxyphenyl)acrylic acid (pure).


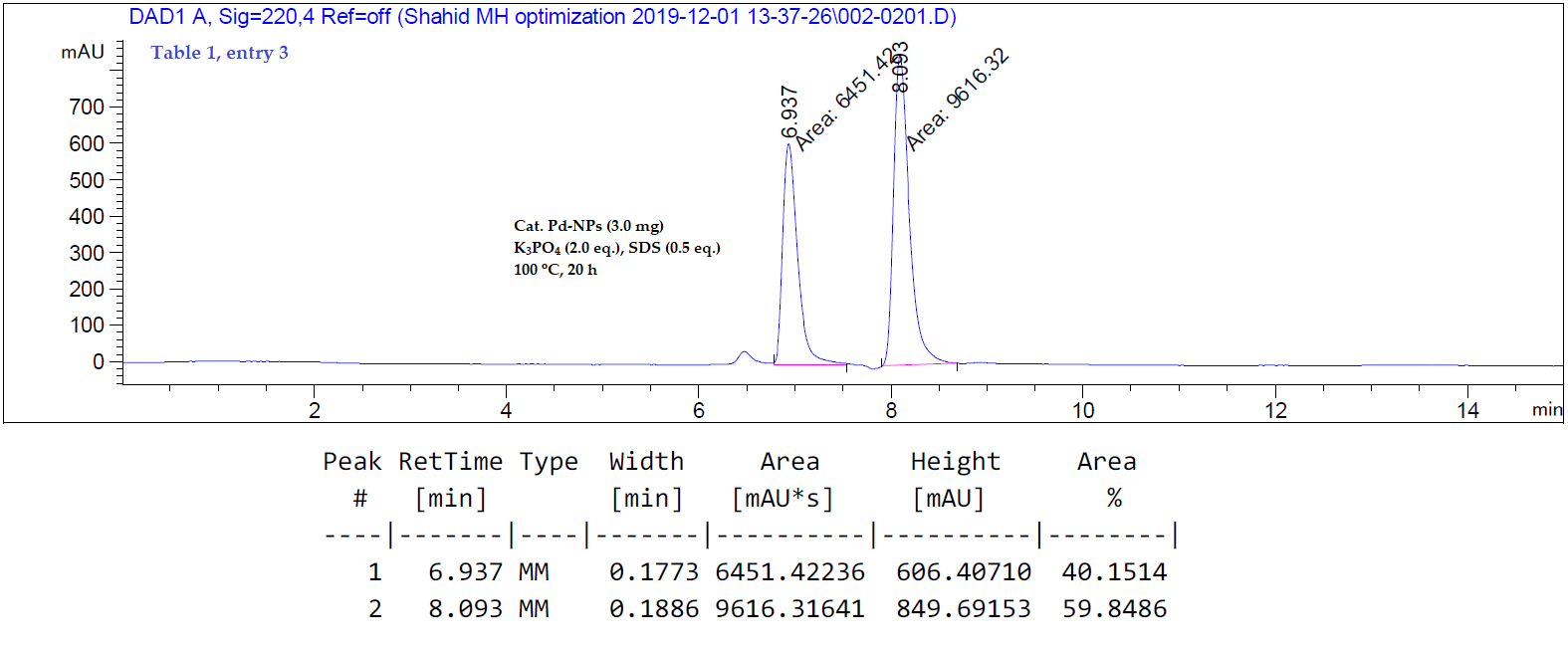


## Supplementary Figure 3. Catalyst screening - Table 1, Entry 3.


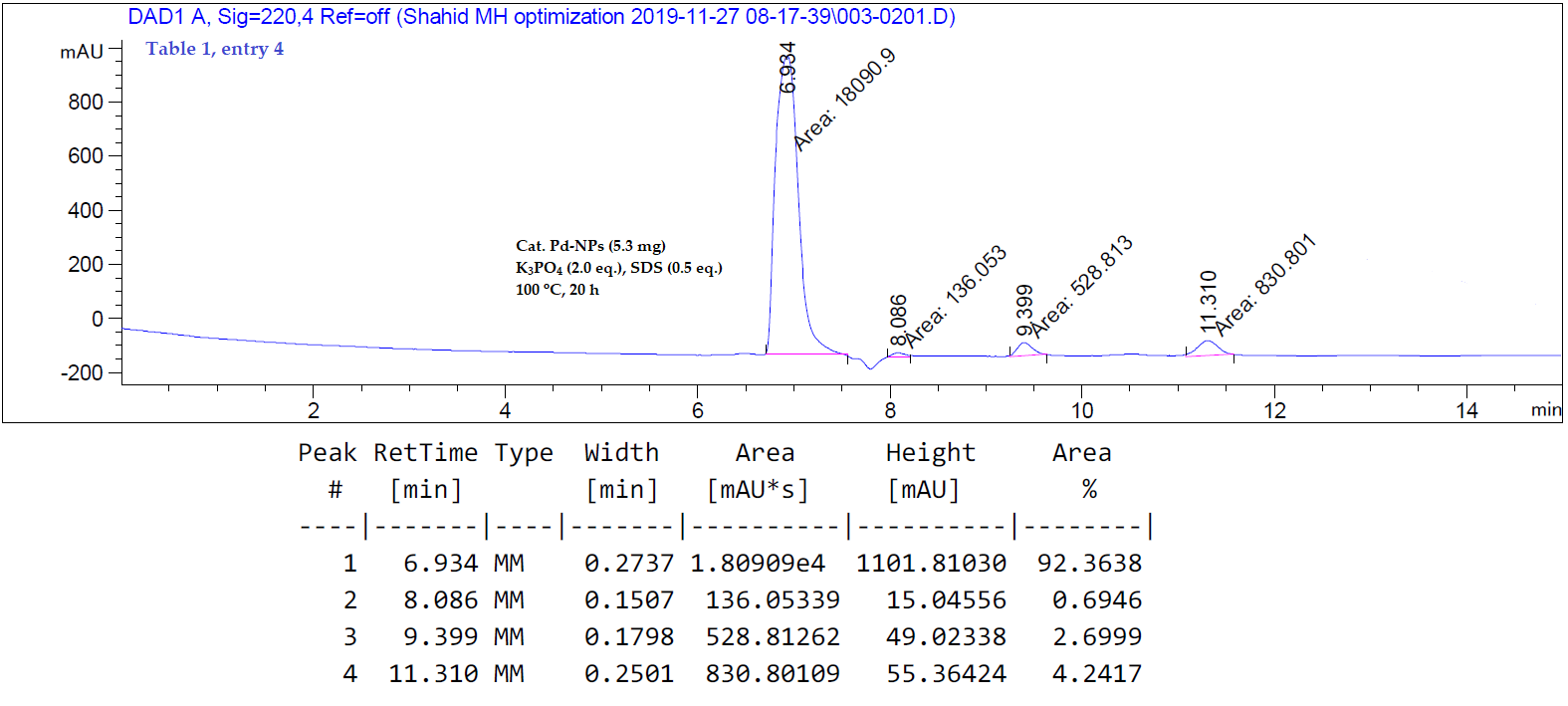


## Supplementary Figure 4. Catalyst screening - Table 1, Entry 4.


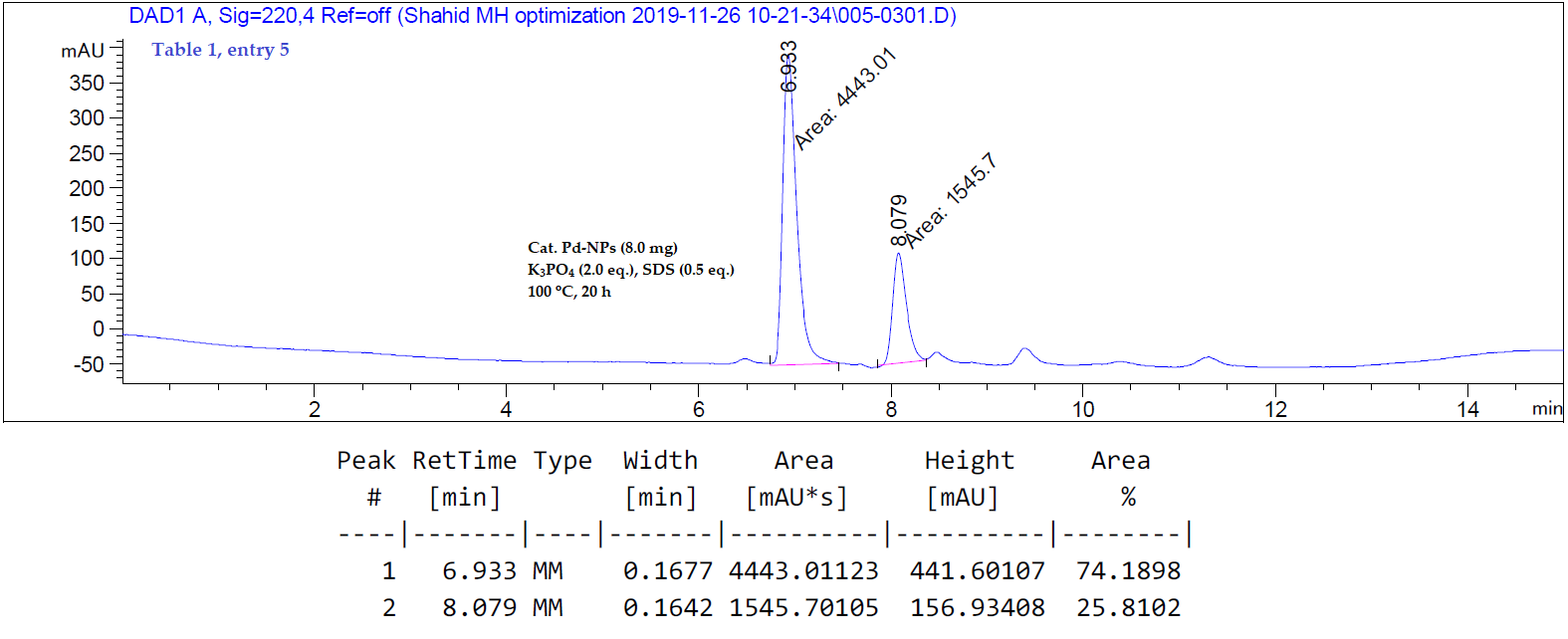


## Supplementary Figure 5. Catalyst screening - Table 1, Entry 5.


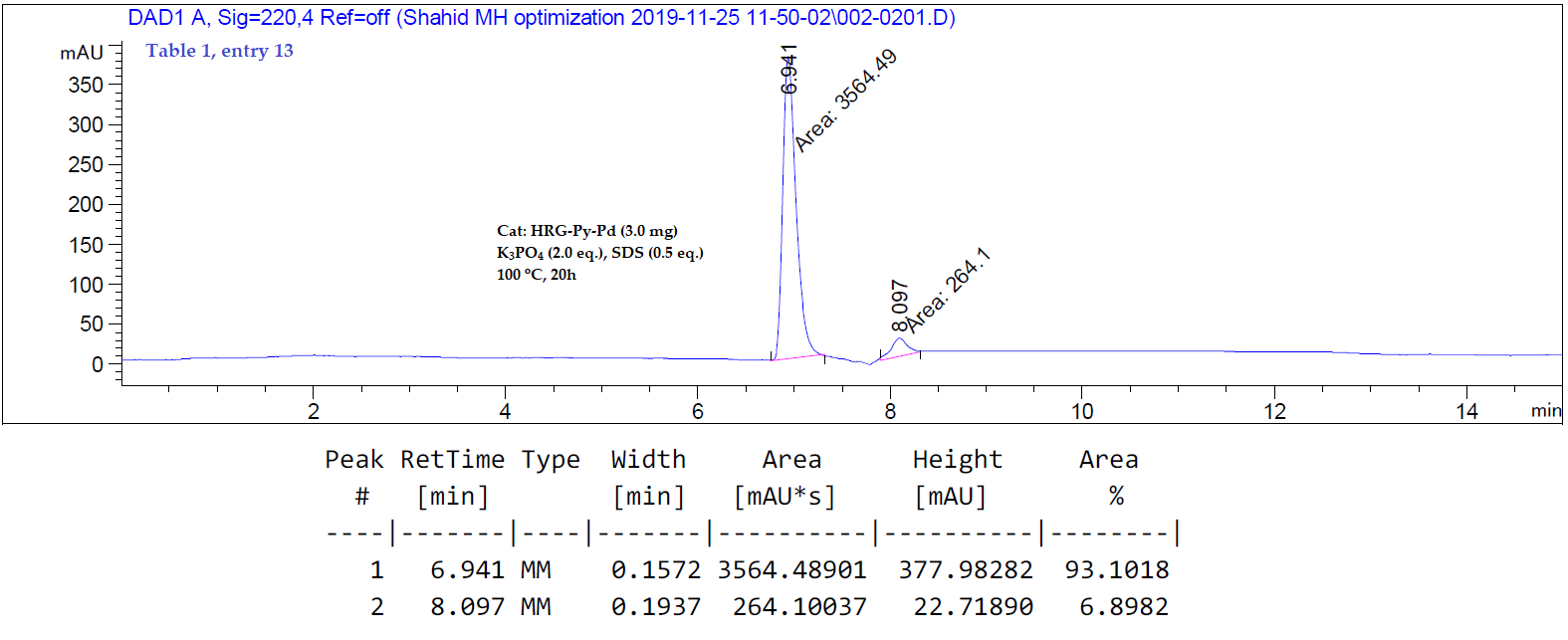


## Supplementary Figure 6. Catalyst screening - Table 1, Entry 13.


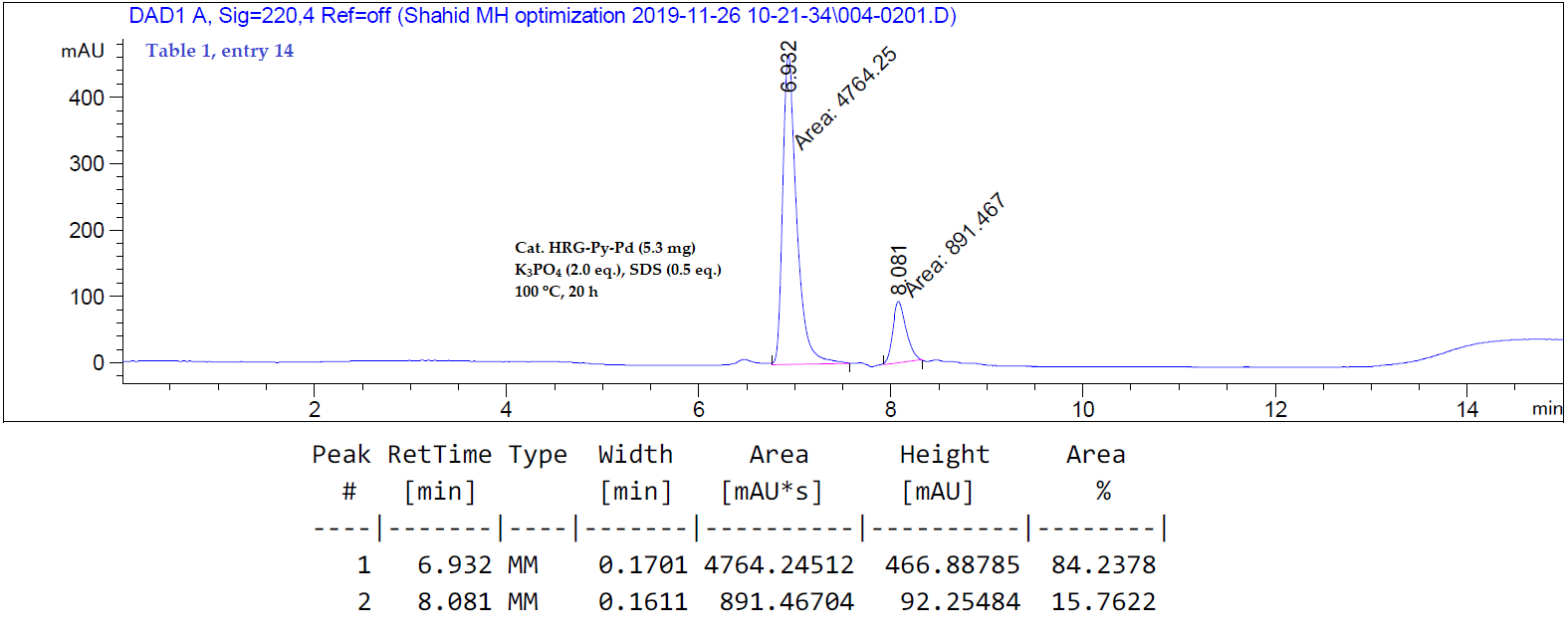


## Supplementary Figure 7. Catalyst screening - Table 1, Entry 14.


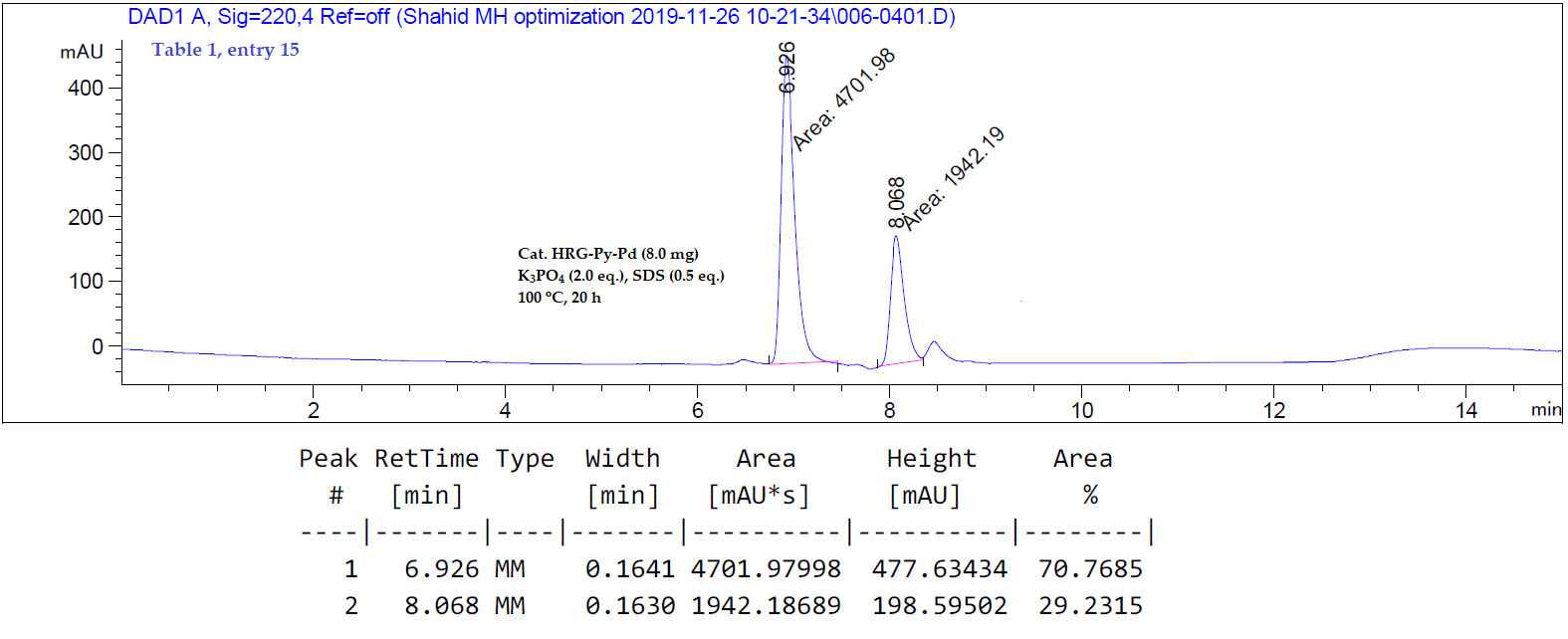


## Supplementary Figure 8. Catalyst screening - Table 1, Entry 15.


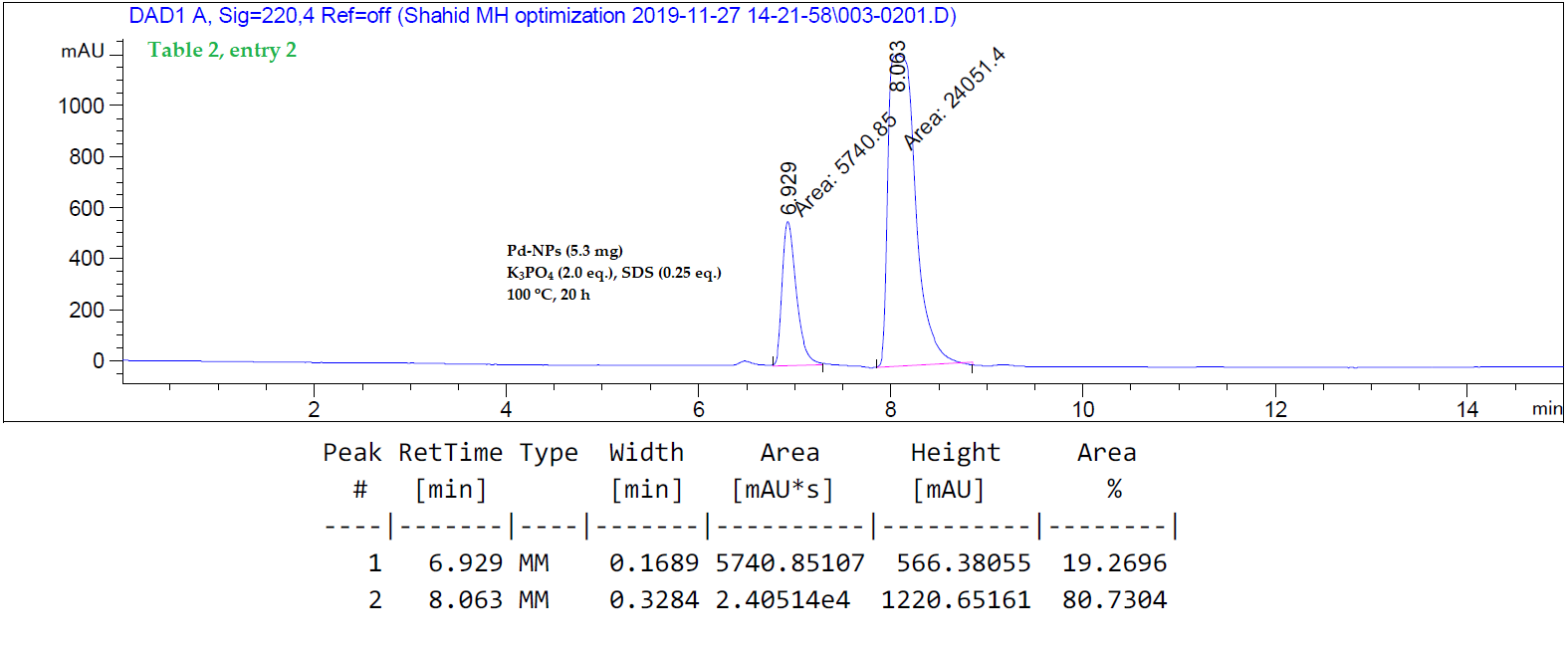


## Supplementary Figure 9. Reagent and time optimization- Table 2, Entry 2.


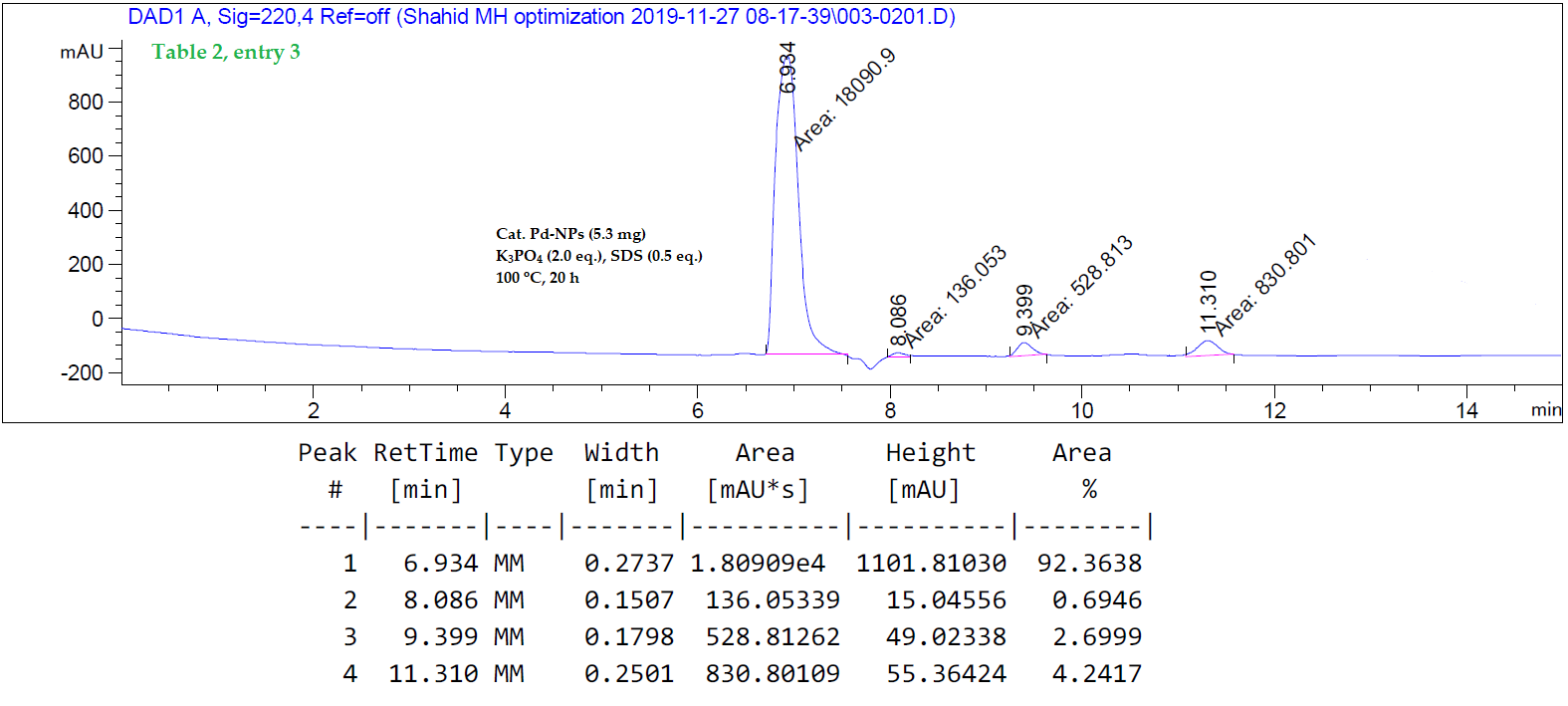


## Supplementary Figure 10. Reagent and time optimization- Table 2, Entry 3.


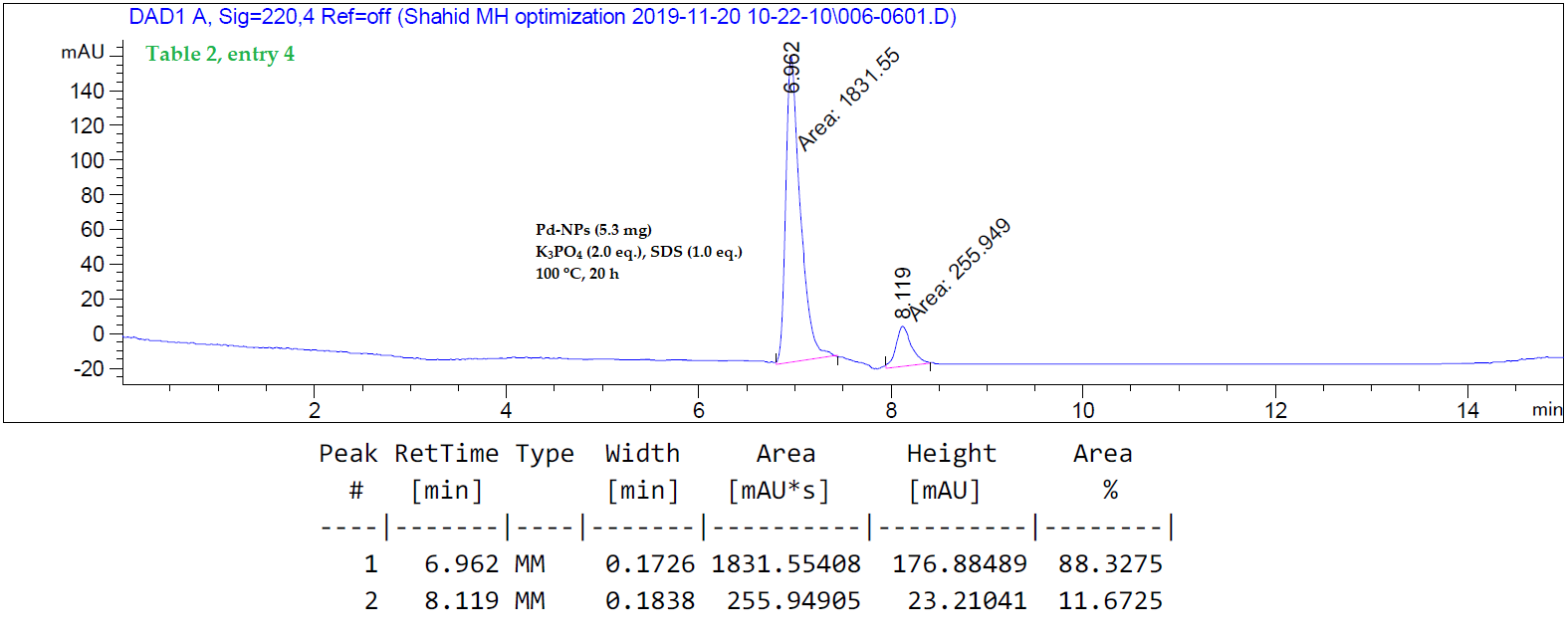


## Supplementary Figure 11. Reagent and time optimization- Table 2, Entry 4.


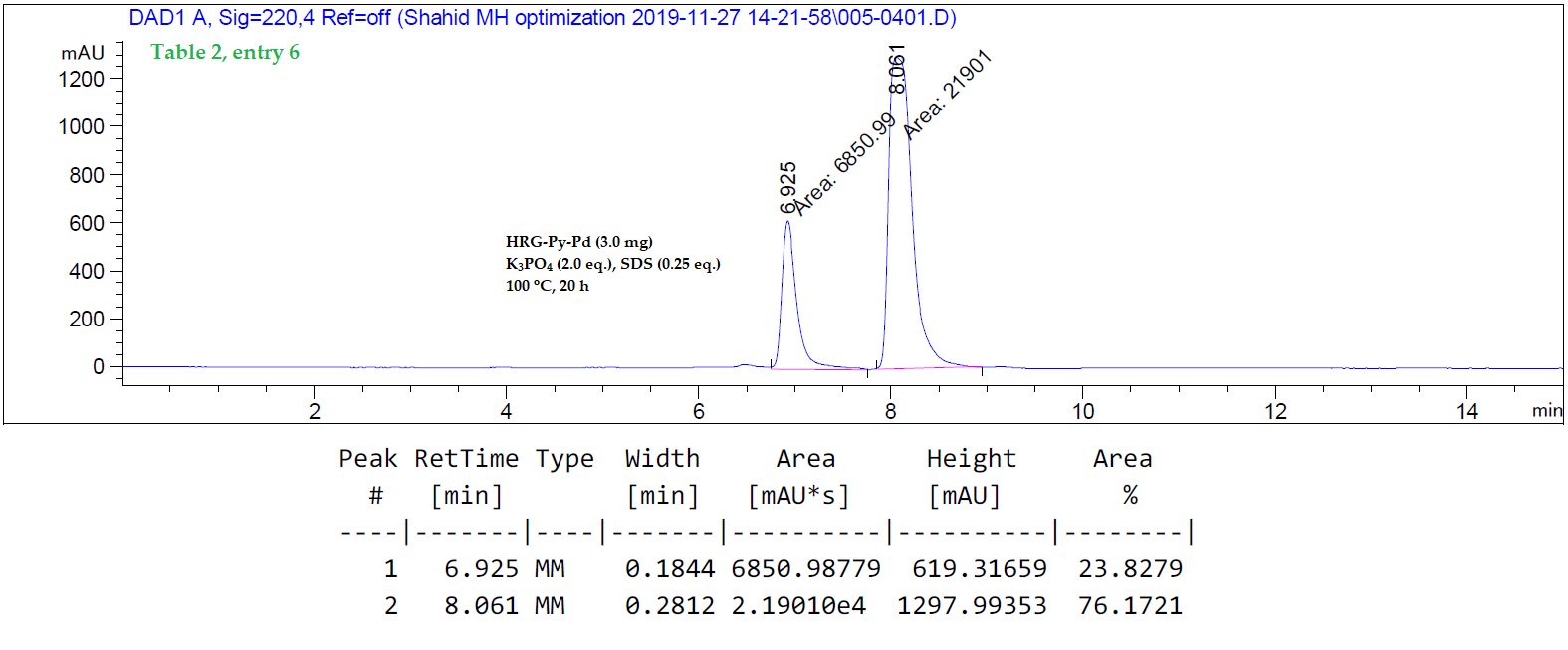


## Supplementary Figure 12. Reagent and time optimization- Table 2, Entry 6.


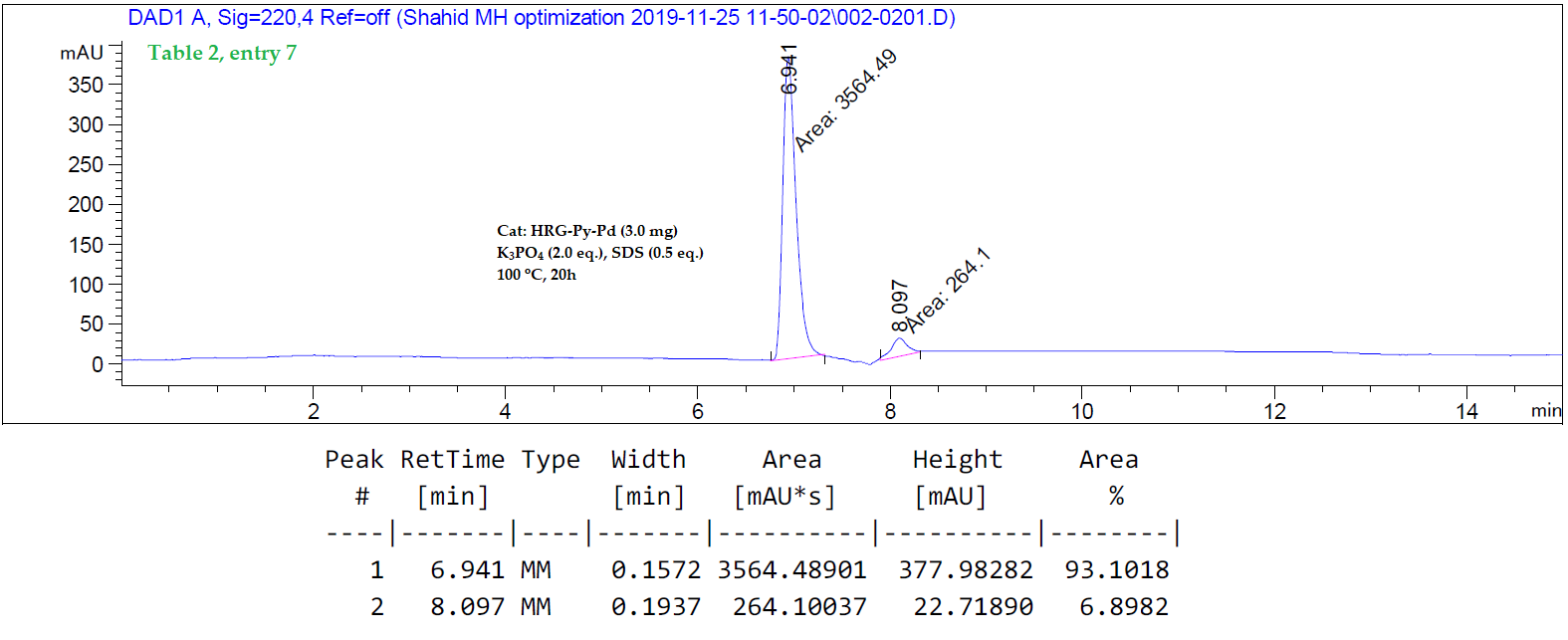


## Supplementary Figure 13. Reagent and time optimization- Table 2, Entry 7.


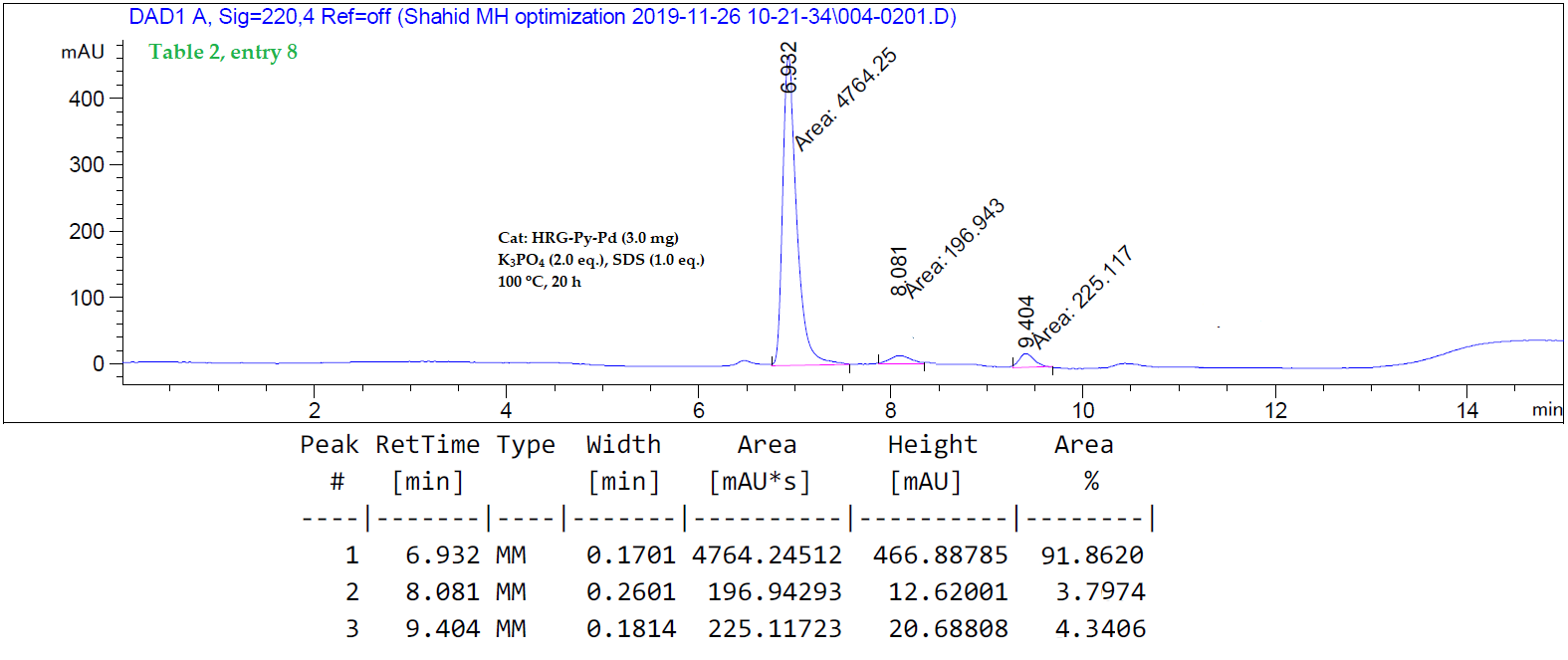


## Supplementary Figure 14. Reagent and time optimization- Table 2, Entry 8.


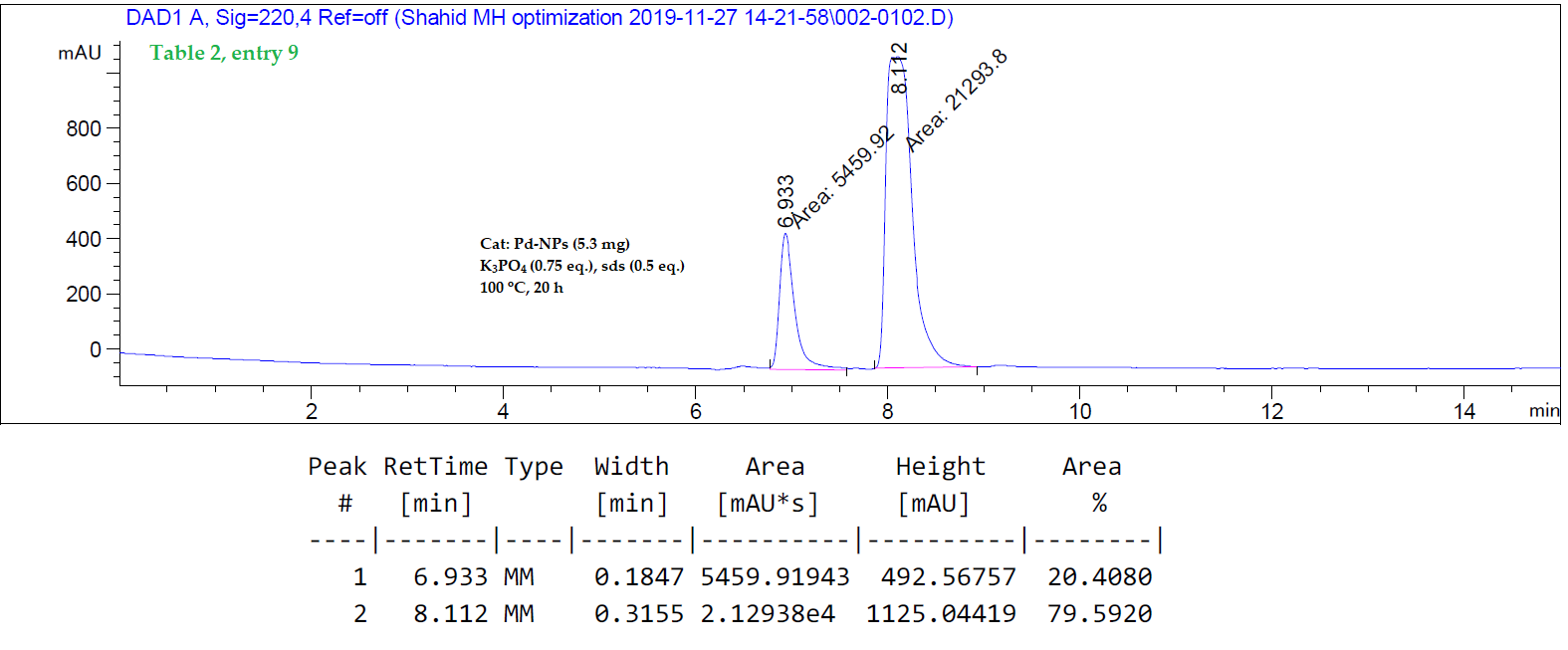


## Supplementary Figure 15. Reagent and time optimization- Table 2, Entry 9.


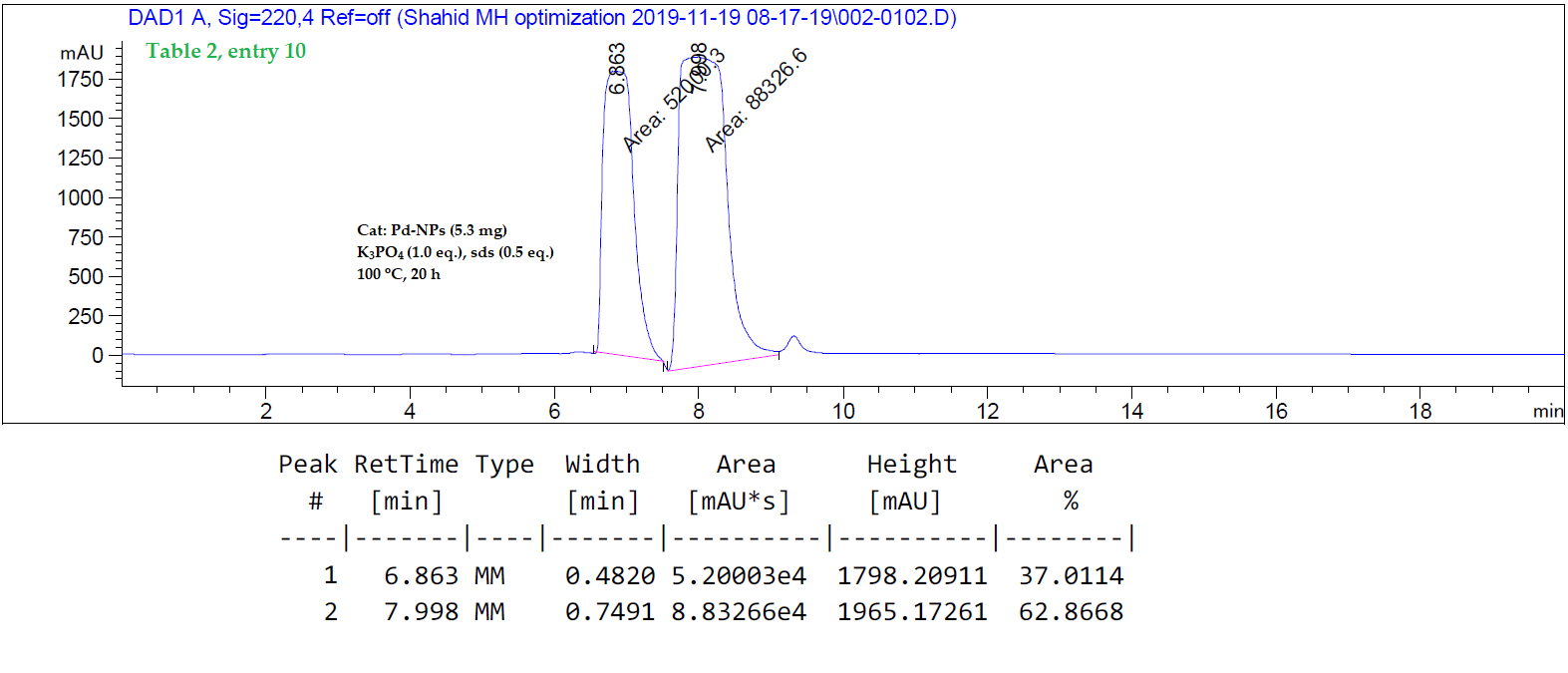


## Supplementary Figure 16. Reagent and time optimization- Table 2, Entry 10.


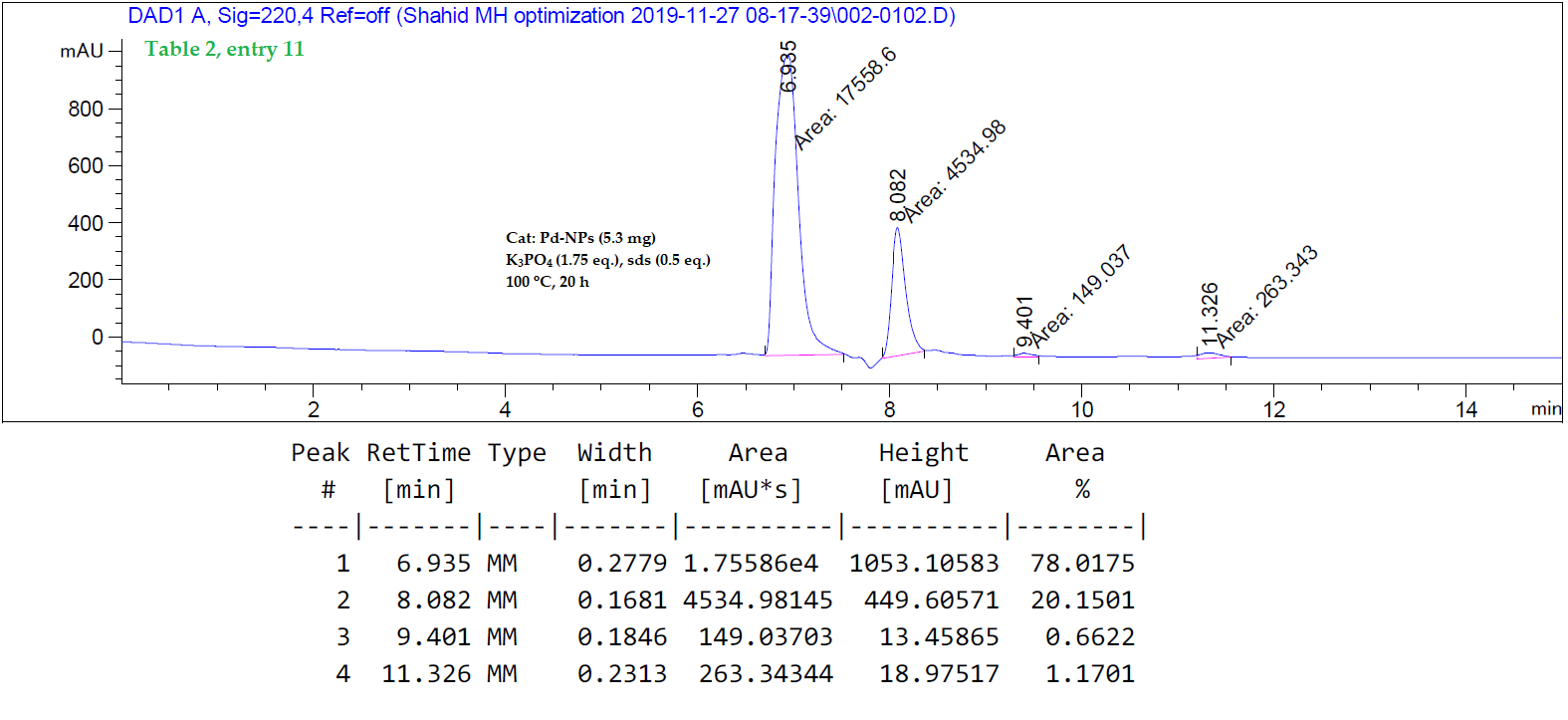


## Supplementary Figure 17. Reagent and time optimization- Table 2, Entry 11.


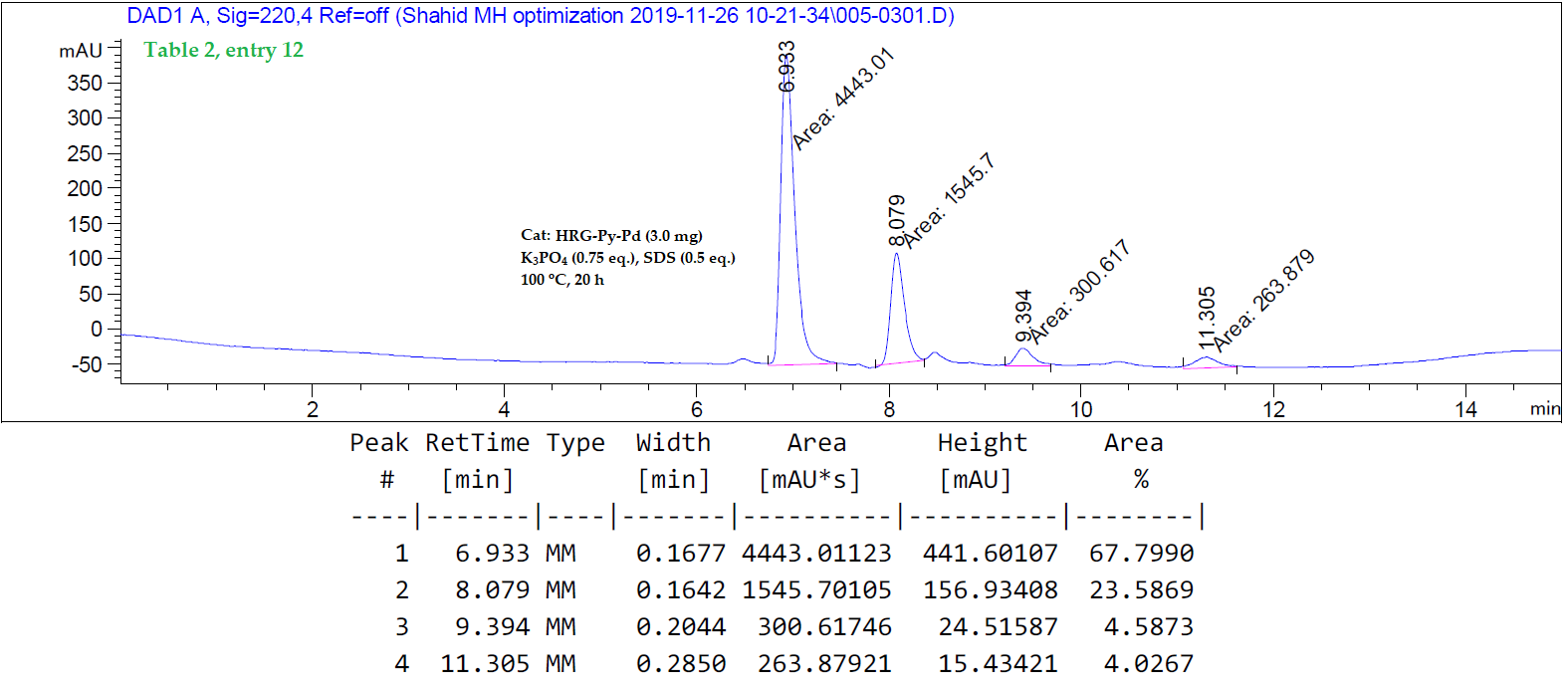


## Supplementary Figure 18. Reagent and time optimization- Table 2, Entry 12.


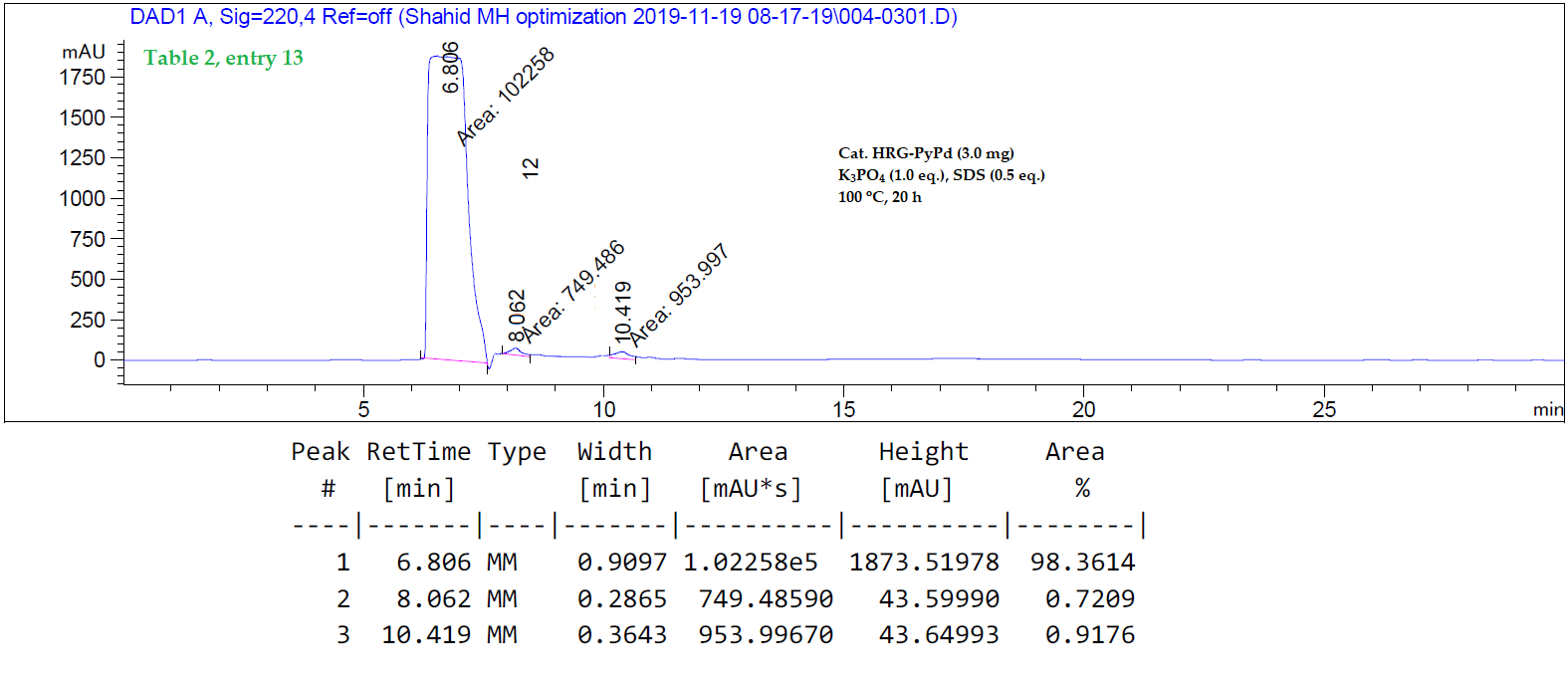


## Supplementary Figure 19. Reagent and time optimization- Table 2, Entry 13.


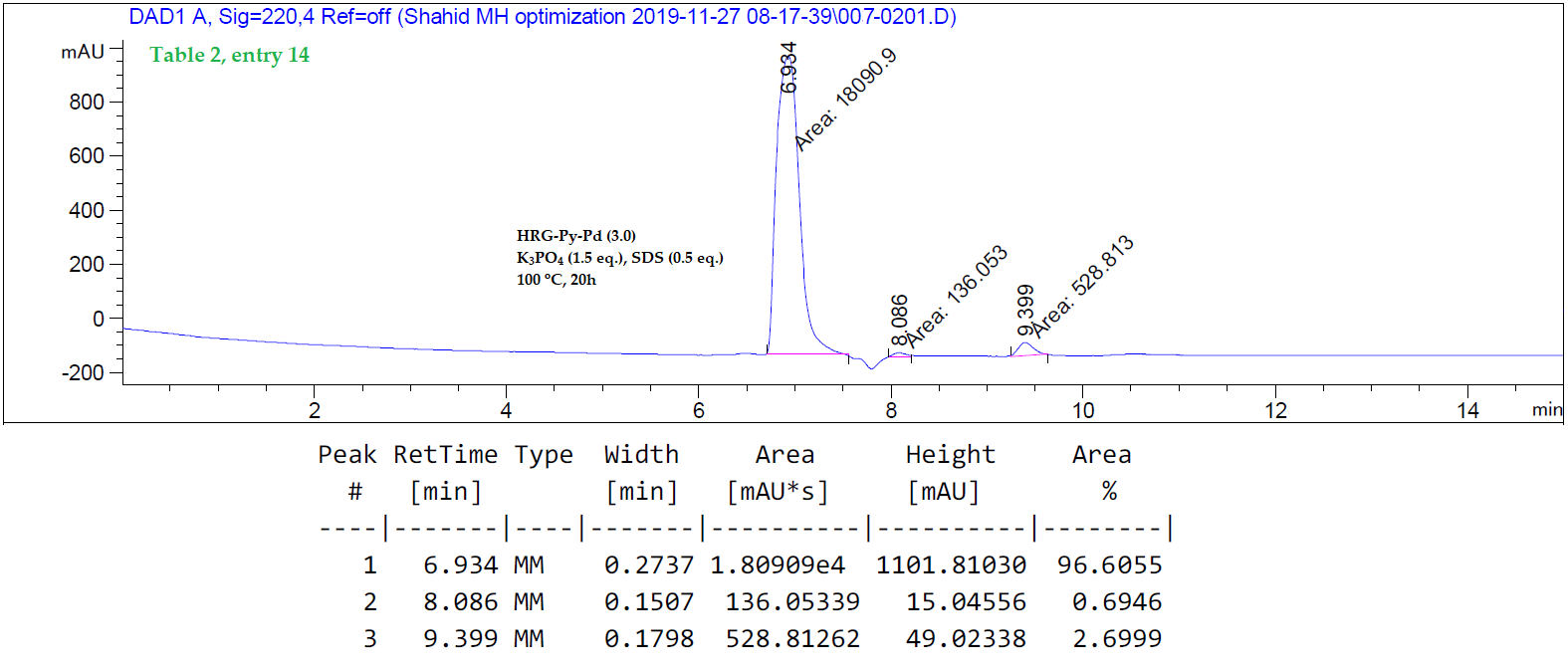


## Supplementary Figure 20. Reagent and time optimization- Table 2, Entry 14.


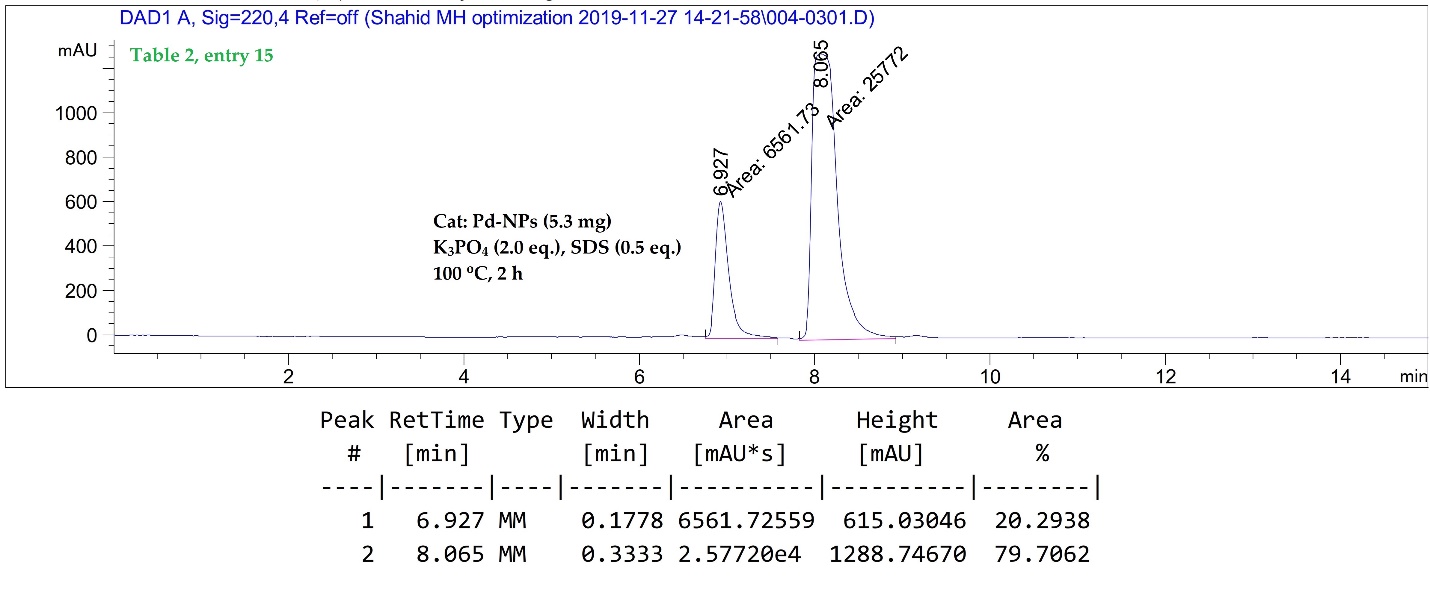


## Supplementary Figure 21. Reagent and time optimization- Table 2, Entry 15.


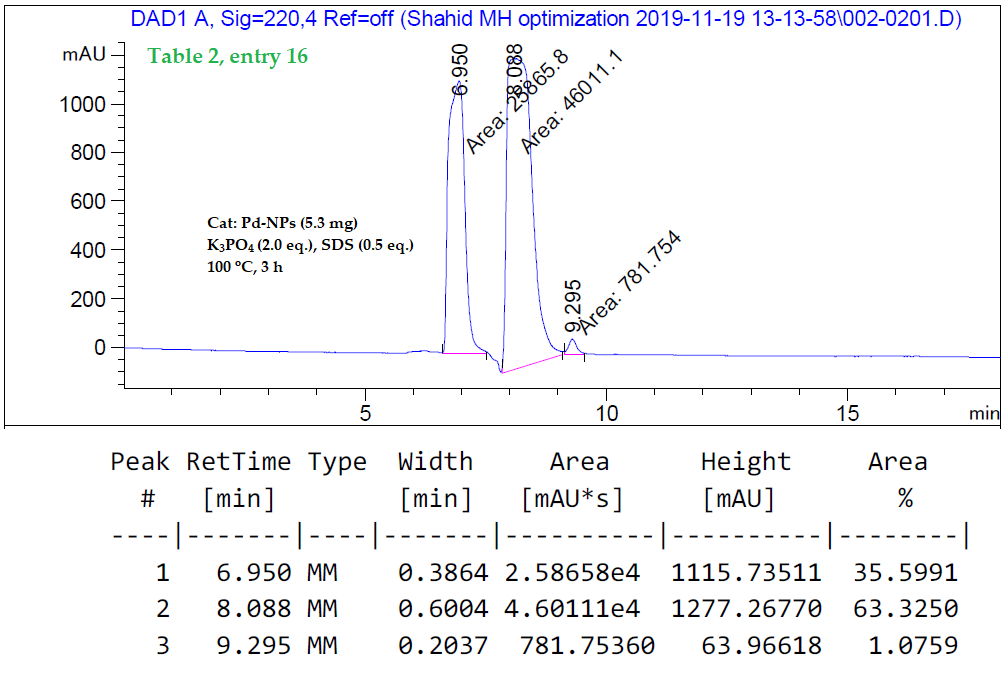


## Supplementary Figure 22. Reagent and time optimization- Table 2, Entry 16.


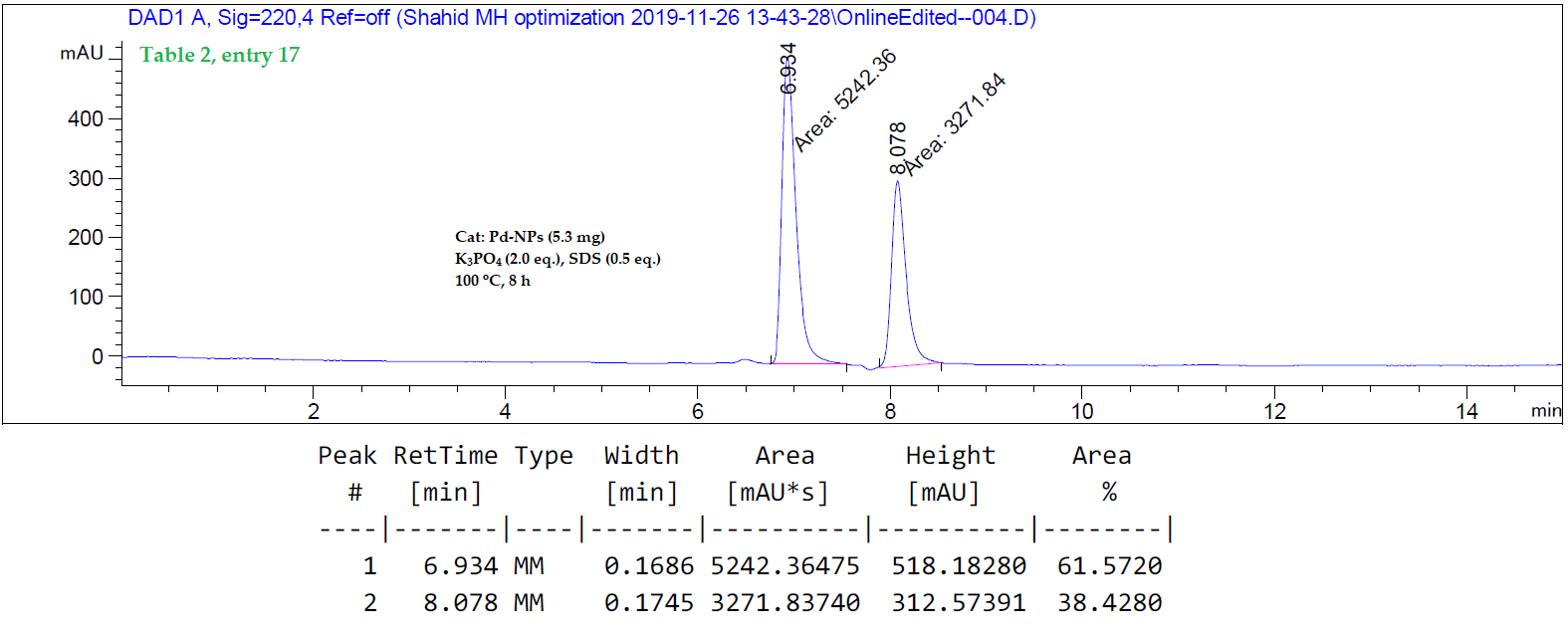


## Supplementary Figure 23. Reagent and time optimization- Table 2, Entry 17.


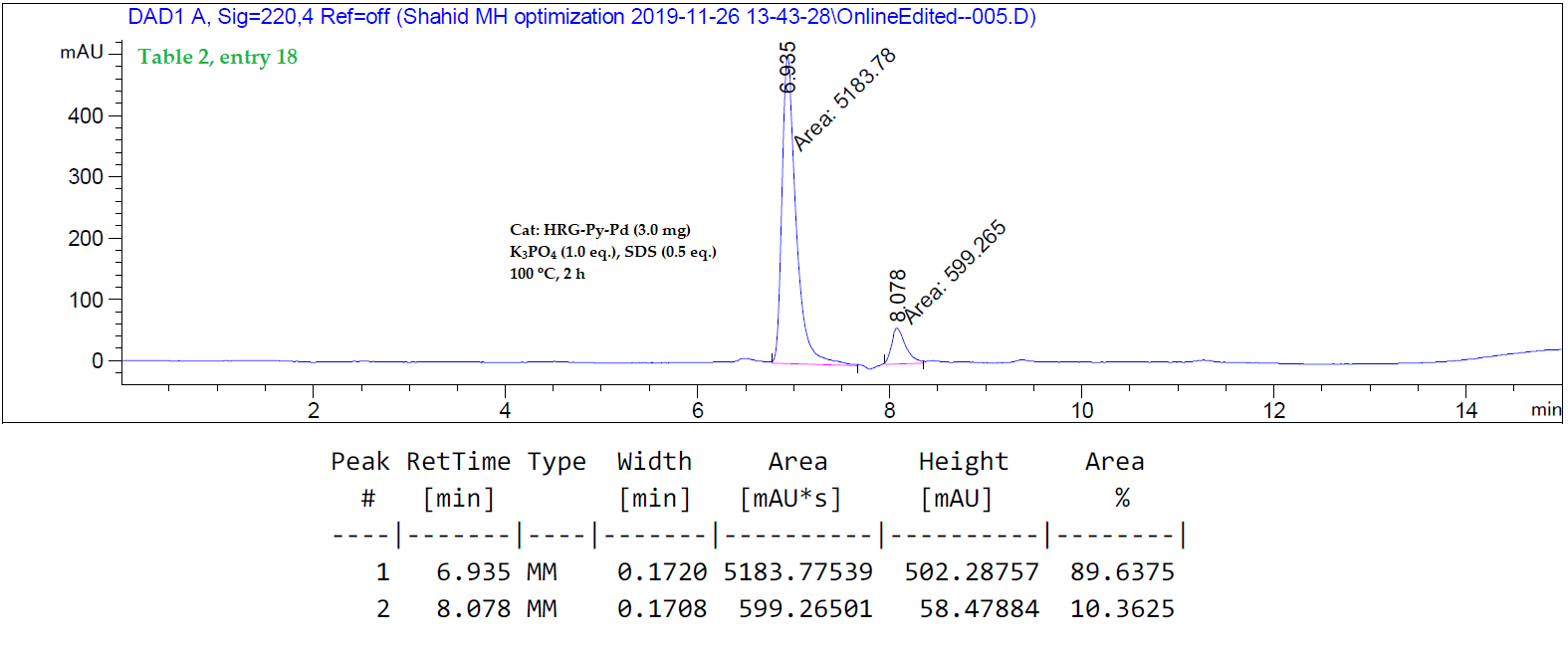


## Supplementary Figure 24. Reagent and time optimization- Table 2, Entry 18.


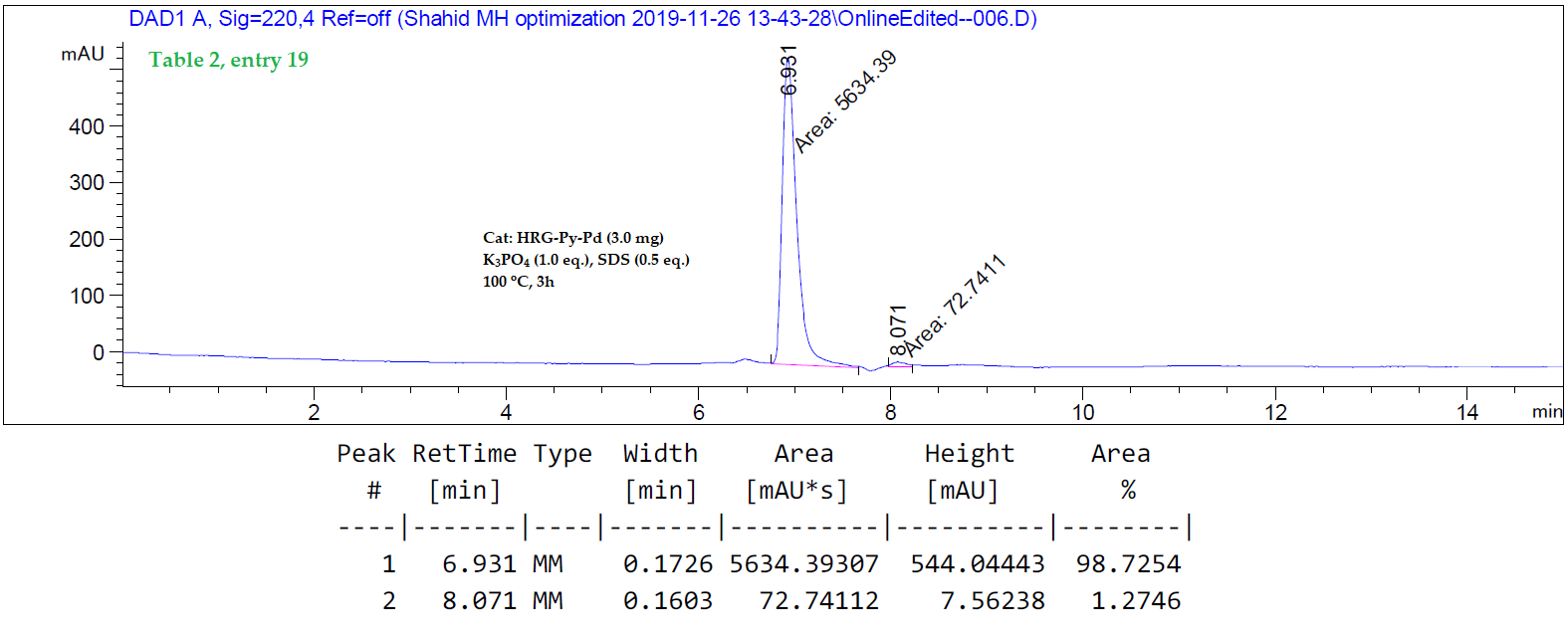


## Supplementary Figure 25. Reagent and time optimization- Table 2, Entry 19.


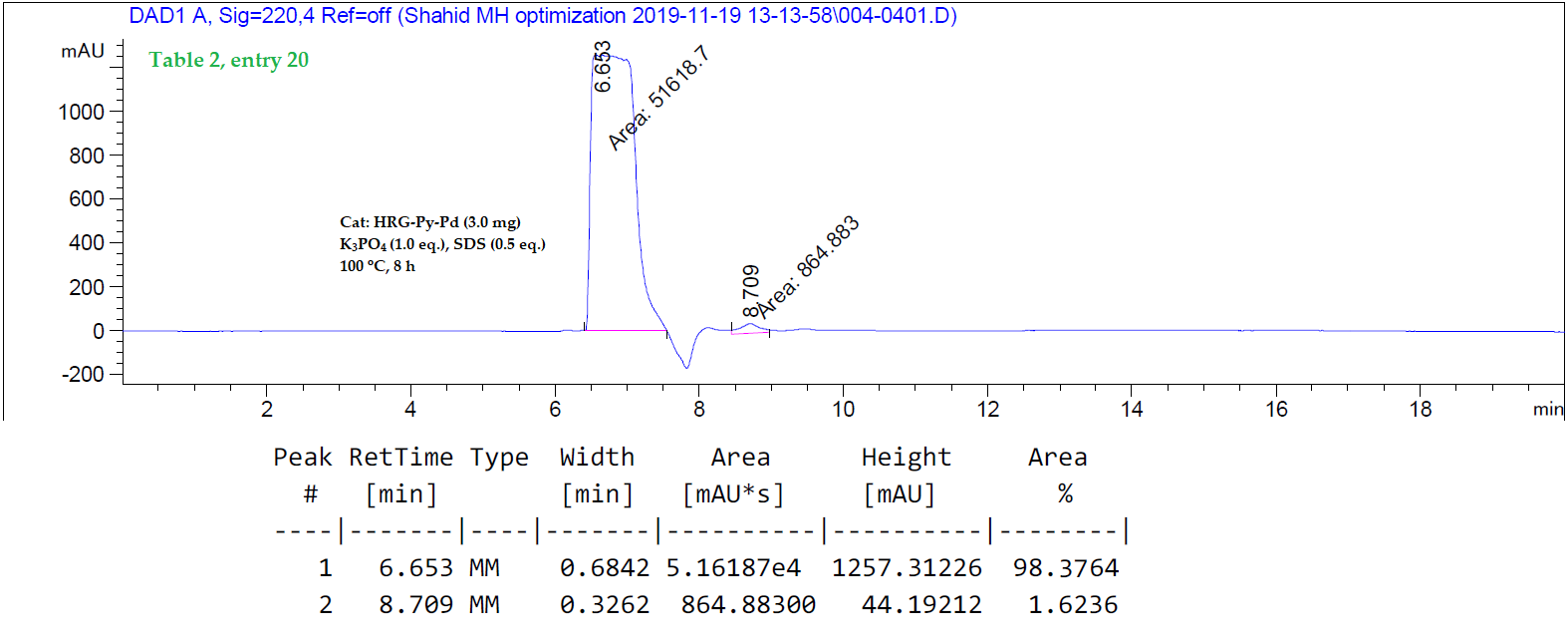


## Supplementary Figure 26. Reagent and time optimization- Table 2, Entry 20.


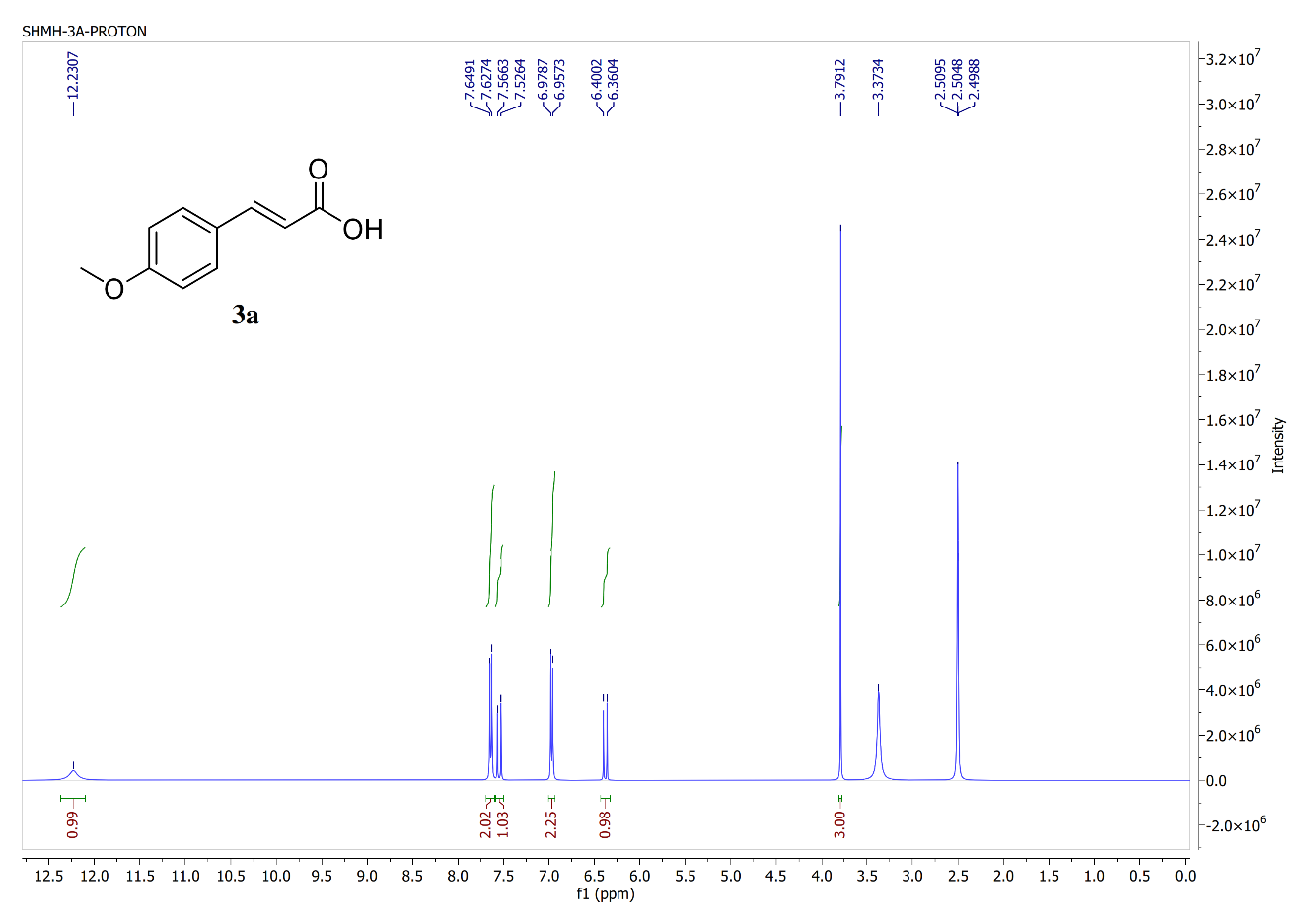


## Supplementary Figure 27. ^1^H-NMR Spectra compound 3a.


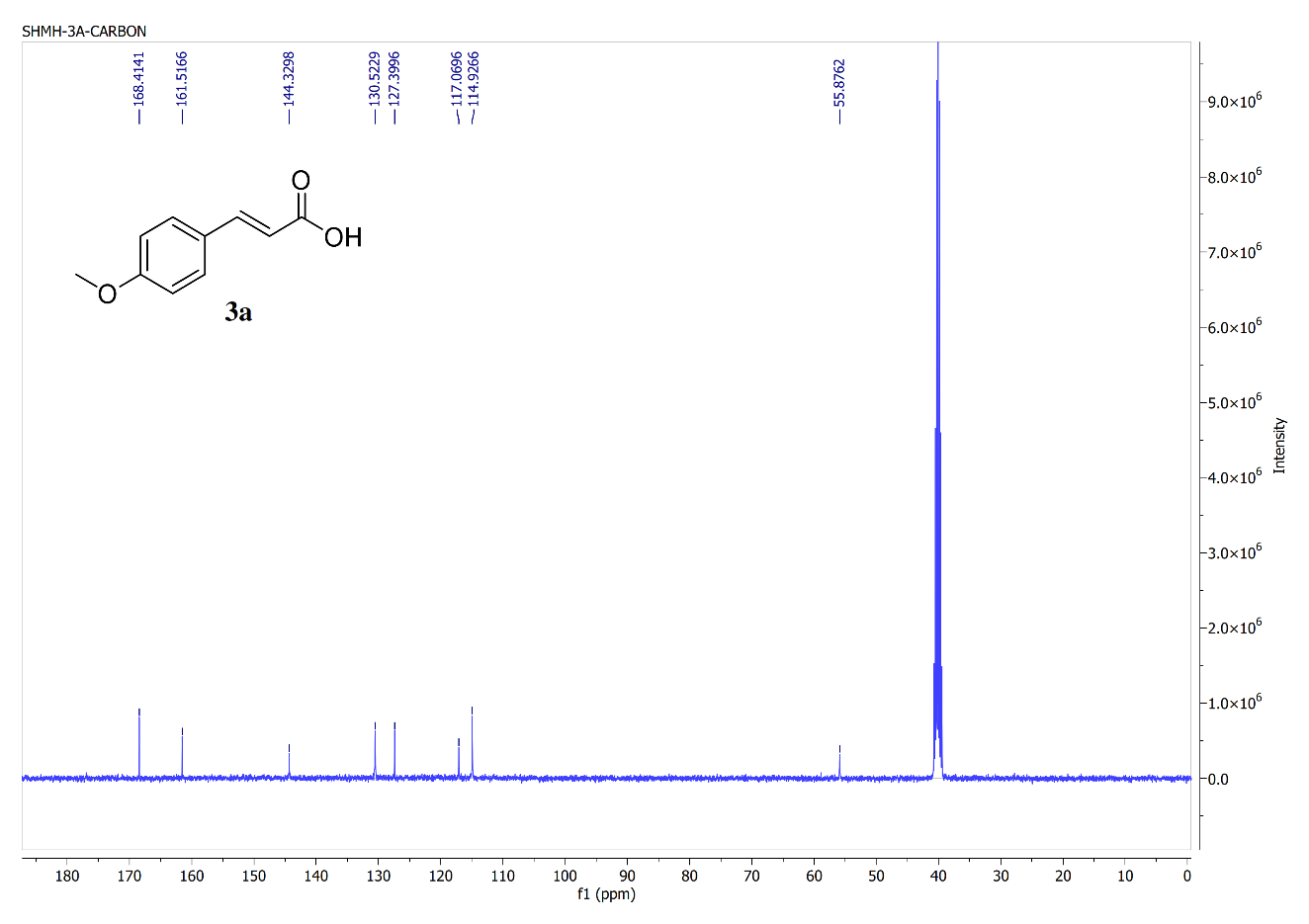


## Supplementary Figure 28. ^13^C-NMR Spectra compound 3a.


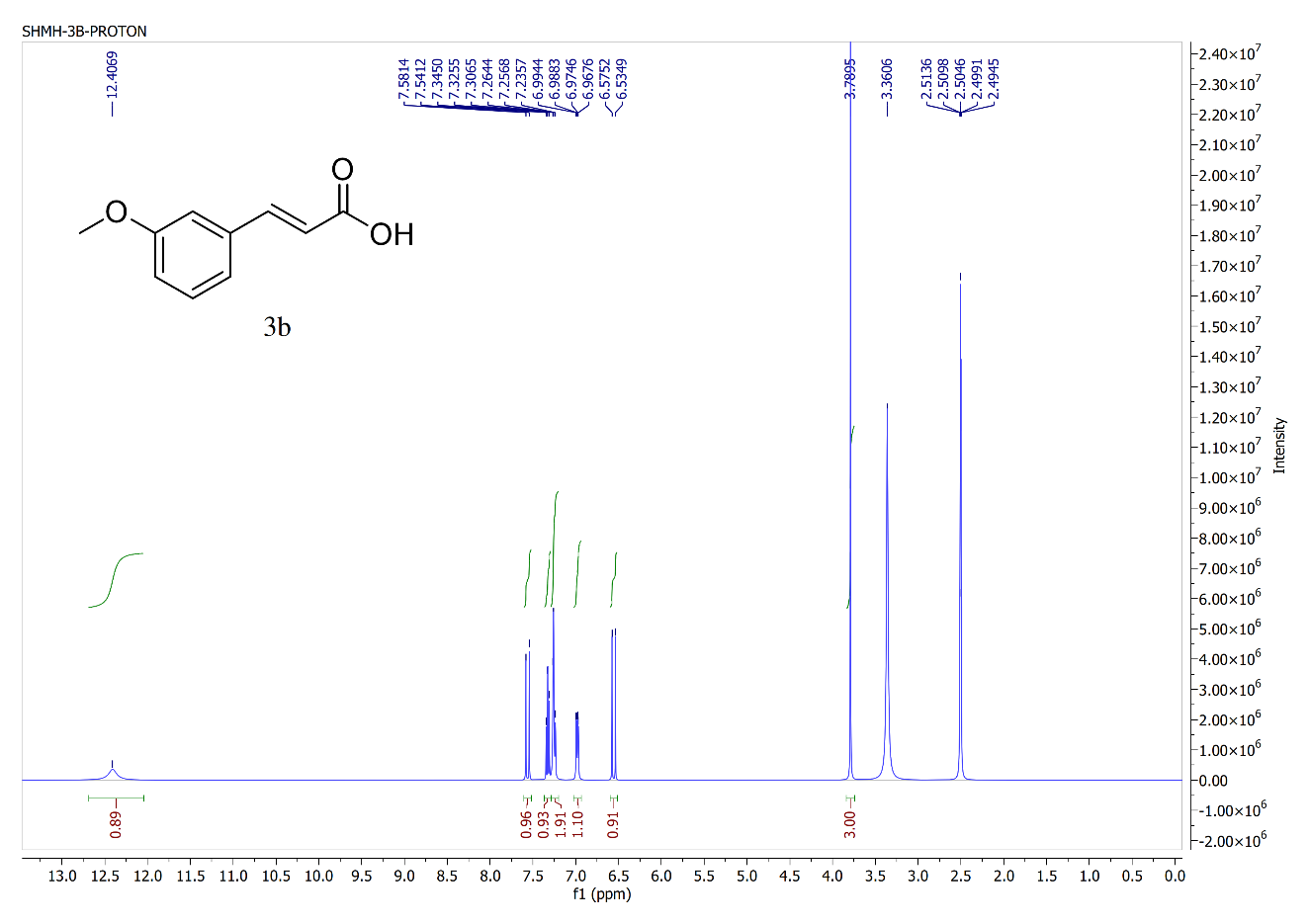


## Supplementary Figure 29. ^1^H-NMR Spectra compound 3b.


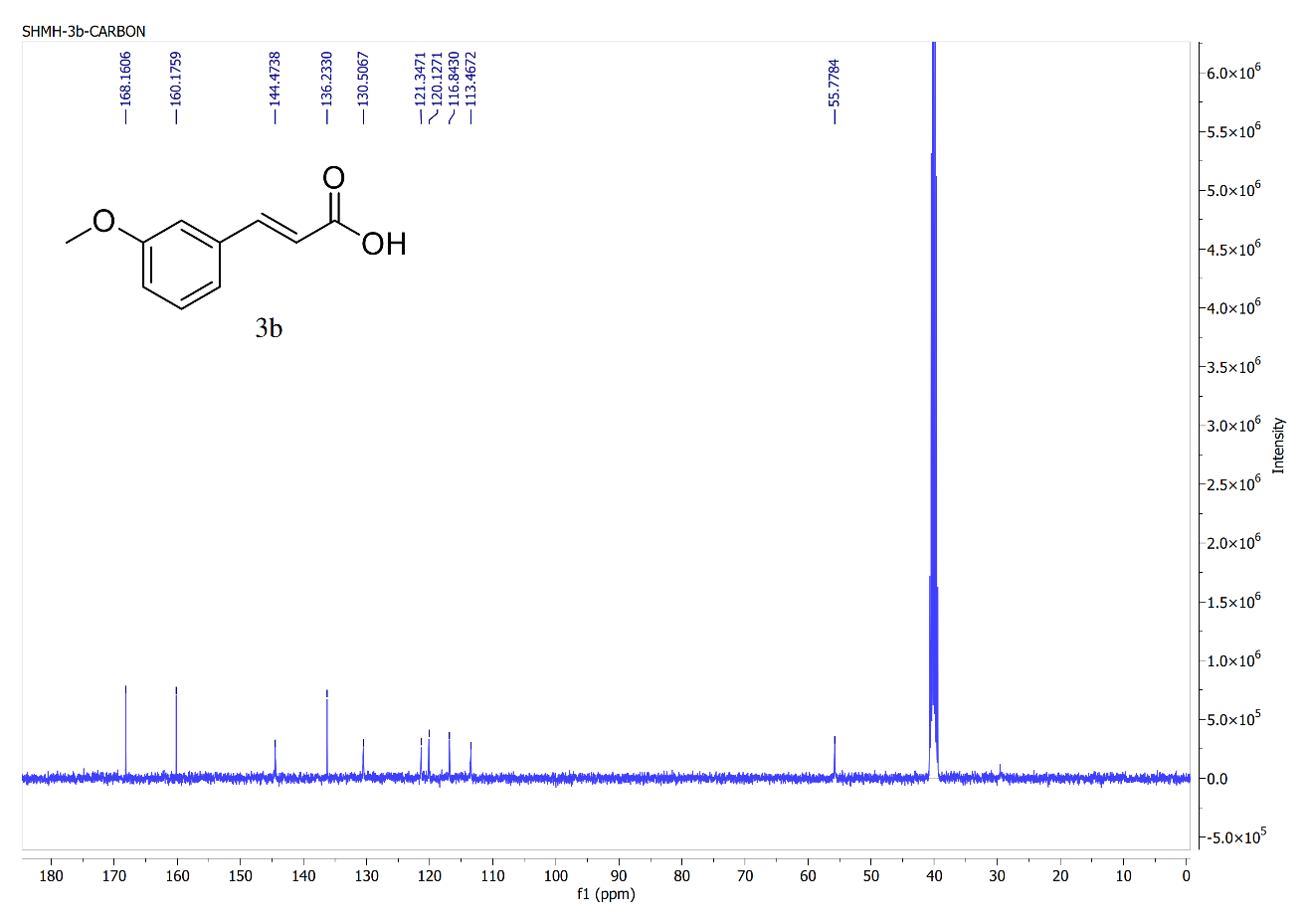


## Supplementary Figure 30. ^13^C-NMR Spectra compound 3b.


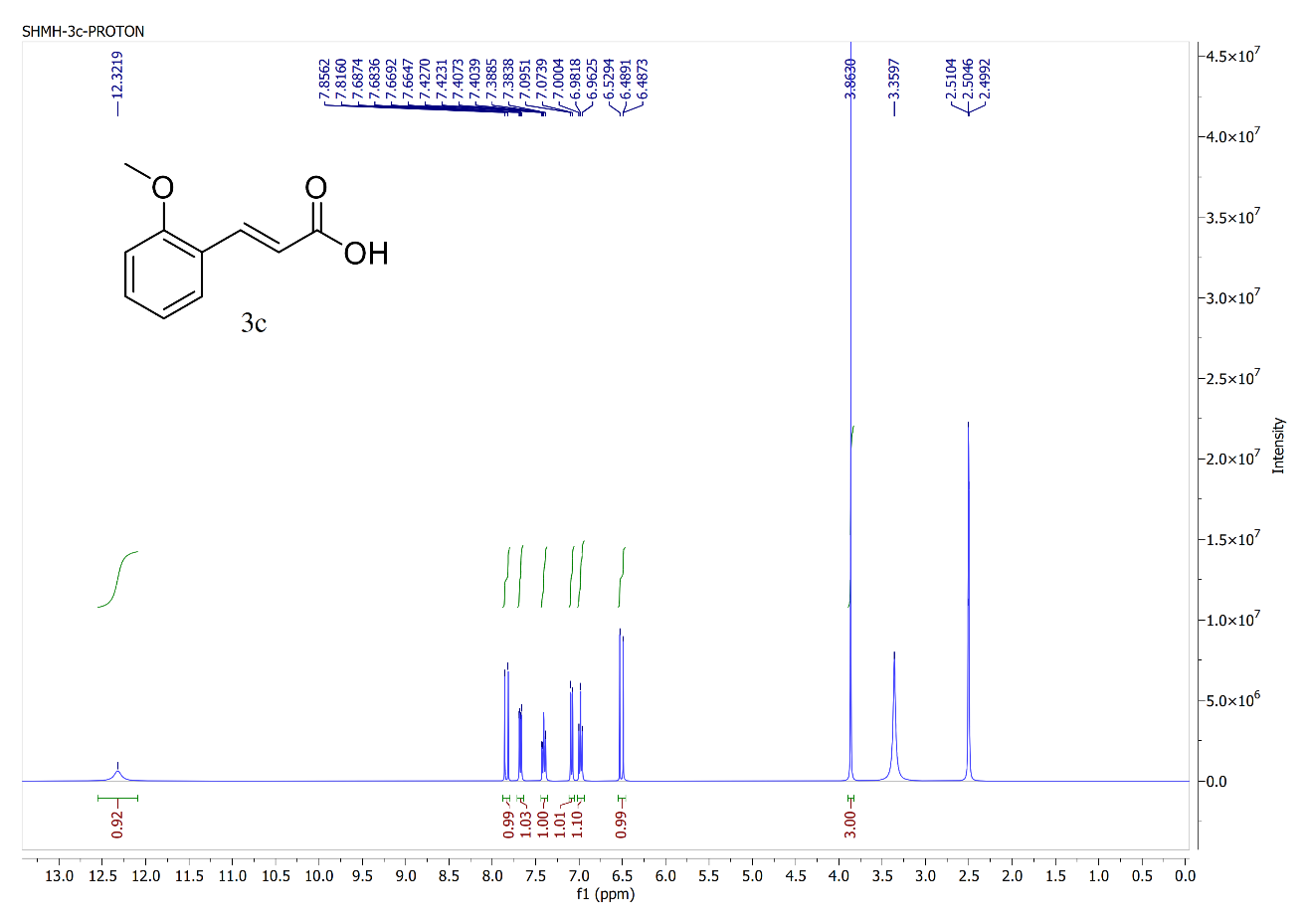


## Supplementary Figure 31. ^1^H-NMR Spectra compound 3c.


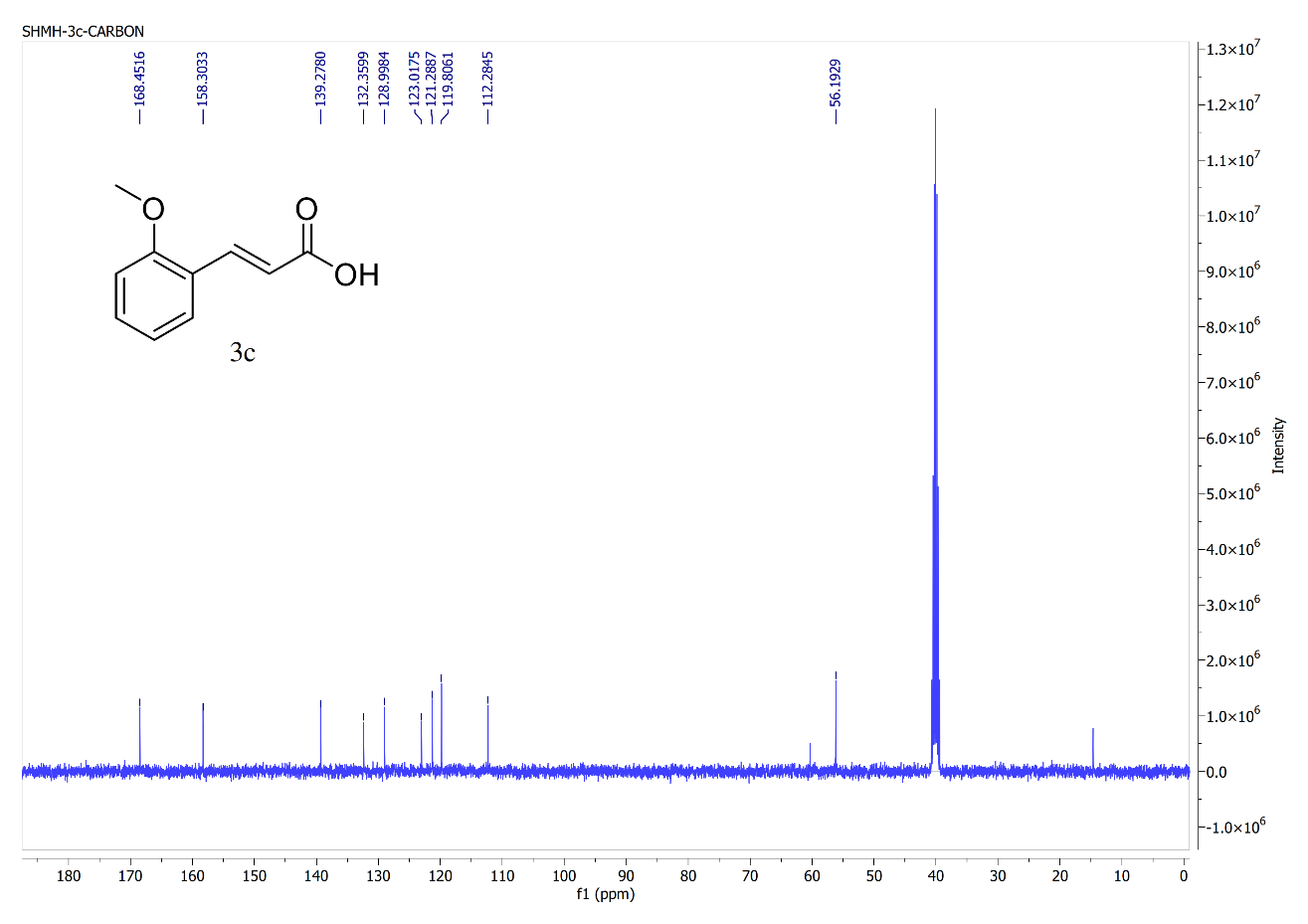


## Supplementary Figure 32. ^13^C-NMR Spectra compound 3c.


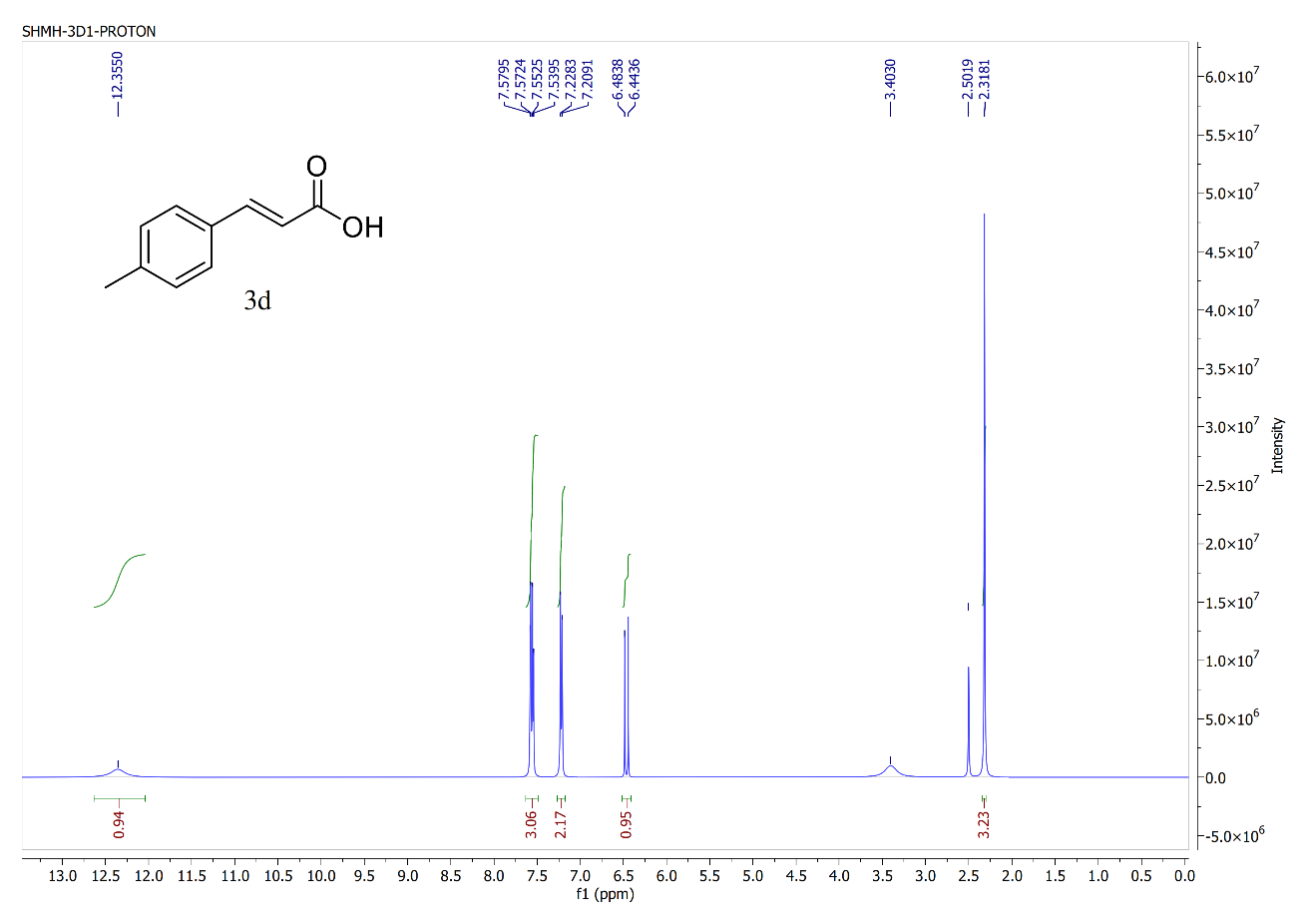


## Supplementary Figure 33. ^1^H-NMR Spectra compound 3d.


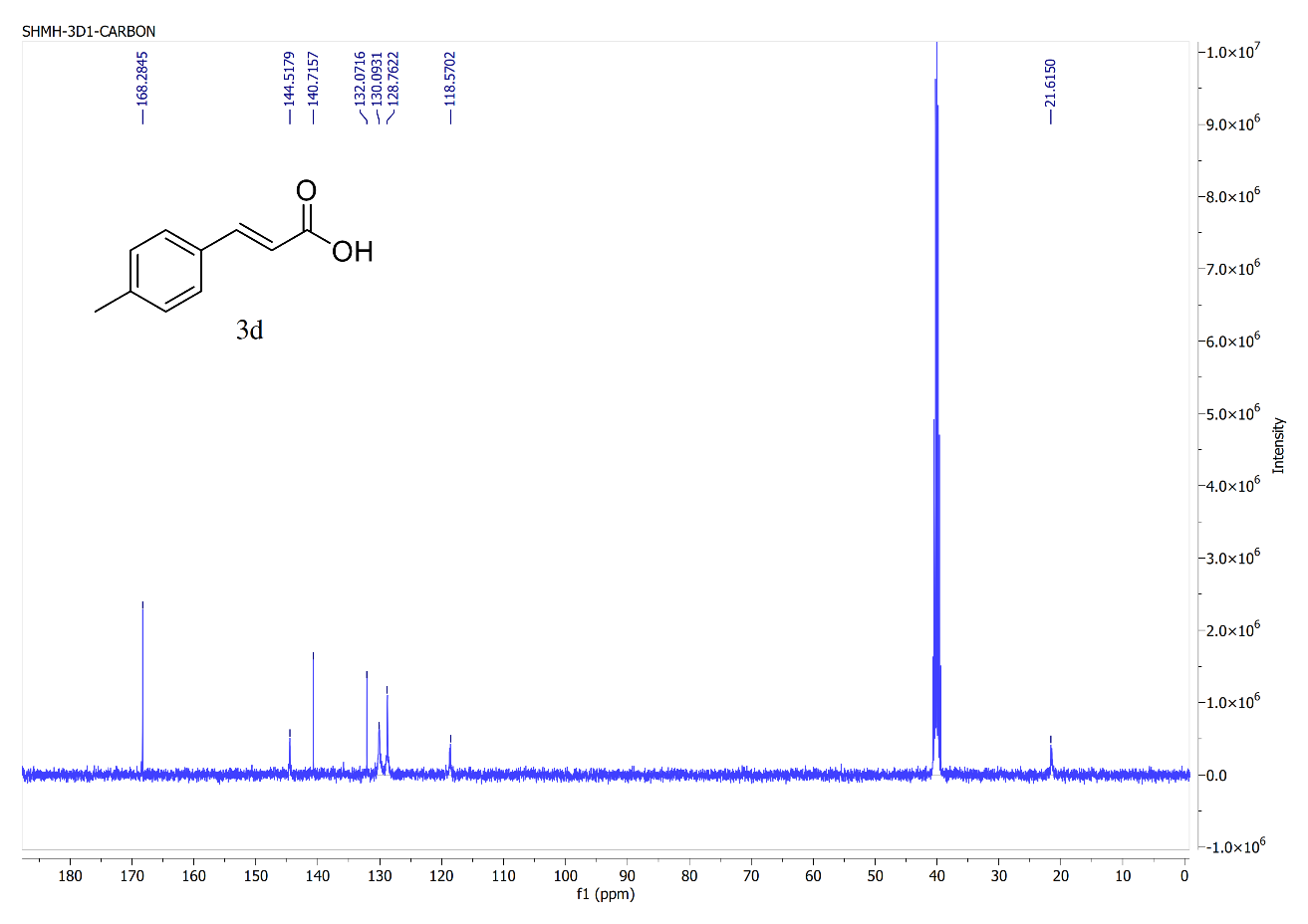


## Supplementary Figure 34. ^13^C-NMR Spectra compound 3d.


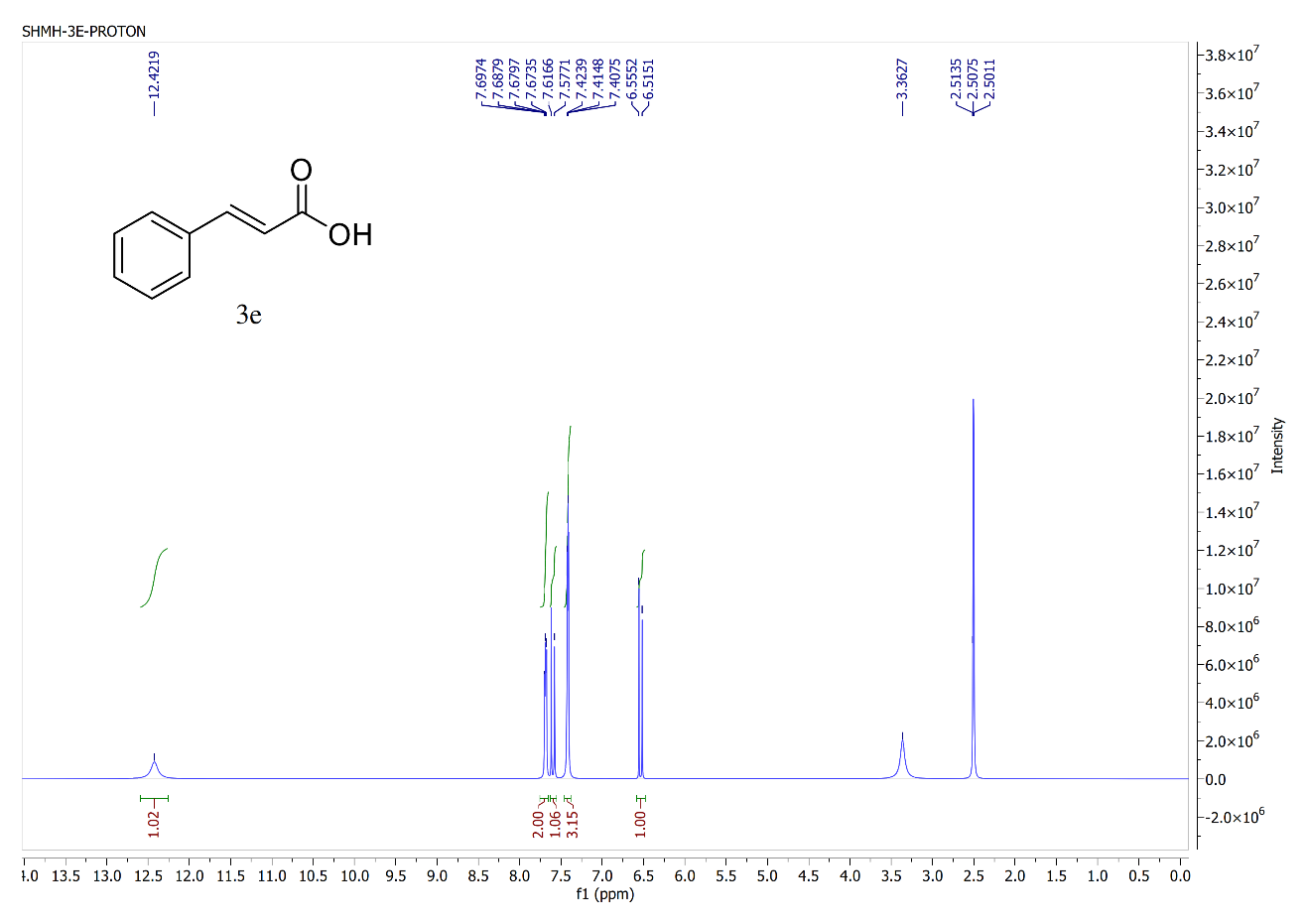


## Supplementary Figure 35. ^1^H-NMR Spectra compound 3e.


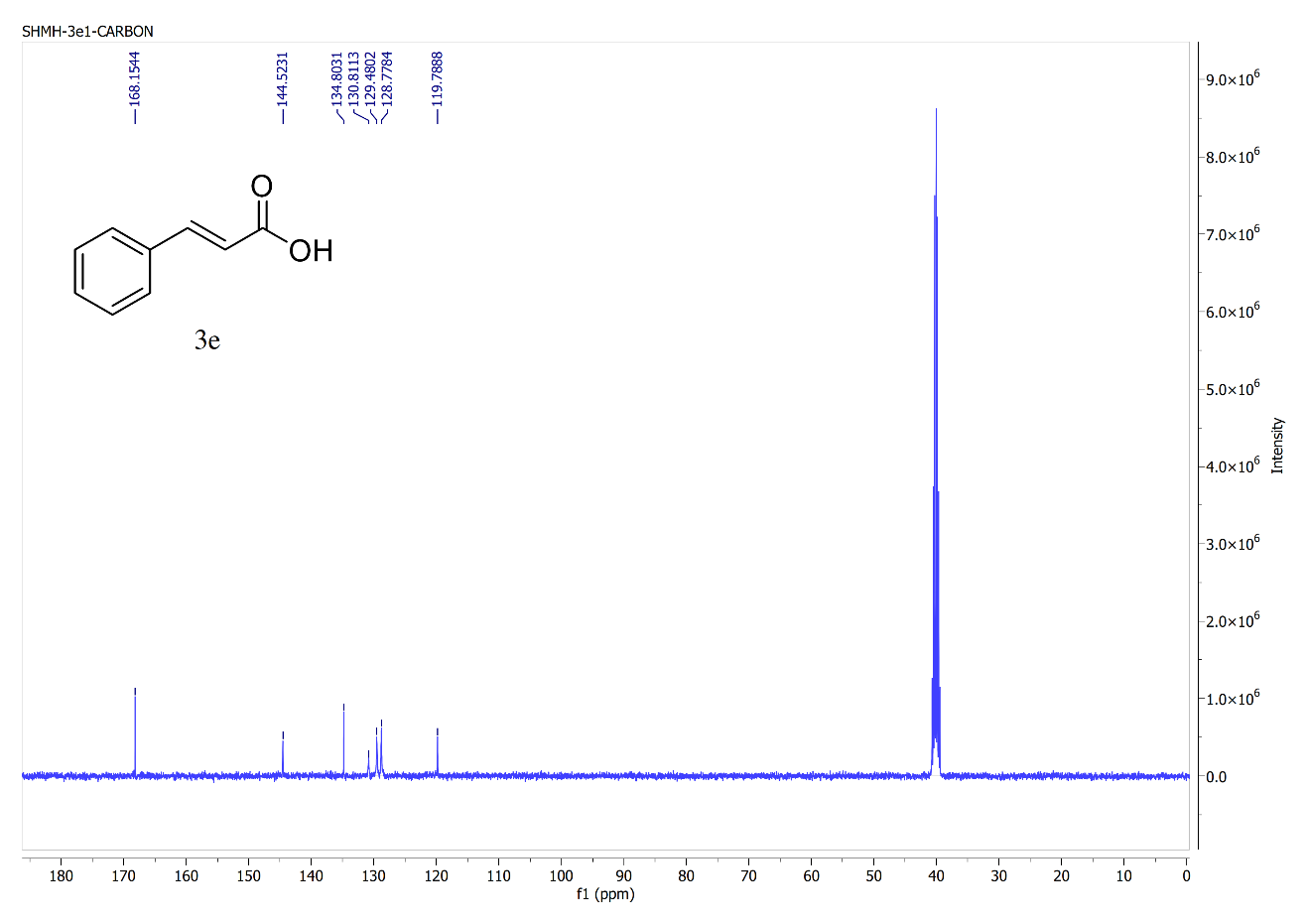


## Supplementary Figure 36. ^13^C-NMR Spectra compound 3e.


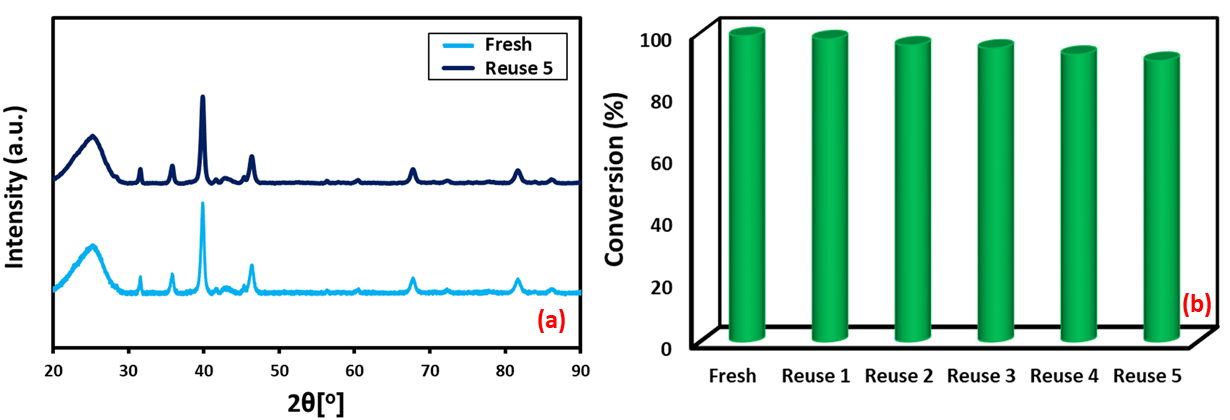


## Supplementary Figure 37. (a) XRD analysis of fresh and reused HRG-Py-Pd nanocatalysts and (b) reusability study of the HRG-Py-Pd nanocatalyst.
